# Disruption of ureide degradation affects plant growth and development during and after transition from vegetative to reproductive stages

**DOI:** 10.1186/s12870-018-1491-2

**Published:** 2018-11-20

**Authors:** Hiroshi Takagi, Shunsuke Watanabe, Shoma Tanaka, Takakazu Matsuura, Izumi C. Mori, Takashi Hirayama, Hiroshi Shimada, Atsushi Sakamoto

**Affiliations:** 1 Graduate School of Science, Hiroshima University, 1-3-1 Kagamiyama, Higashi-Hiroshima, 739-8526 Japan; 2 Institute of Plant Science and Resources, Okayama University, Kurashiki, 710-0046 Japan; 3 Present Address: Microbial and Plant Genomics Institute, University of Minnesota, Saint Paul, MN 55108 USA; 4 Present Address: Center for Sustainable Resource Science, RIKEN, Yokohama, 230-0045 Japan

**Keywords:** Allantoate, Allantoin, *Arabidopsis thaliana*, Catabolic pathway, Nitrogen remobilization, Phytohormone, Purine base, Ureide transport

## Abstract

**Background:**

The ureides allantoin and allantoate are major metabolic intermediates of purine catabolism with high nitrogen-to-carbon ratios. Ureides play a key role in nitrogen utilization in ureide-type legumes, but their effects on growth and development in non-legume plants are poorly understood. Here, we examined the effects of knocking out genes encoding ureide-degrading enzymes, allantoinase (ALN) and allantoate amidohydrolase (AAH), on the vegetative-to-reproductive transition and subsequent growth of Arabidopsis plants.

**Results:**

The ureide-degradation mutants (*aln* and *aah*) showed symptoms similar to those of nitrogen deficiency: early flowering, reduced size at maturity, and decreased fertility. Consistent with these phenotypes, carbon-to-nitrogen ratios and nitrogen-use efficiencies were significantly decreased in ureide-degradation mutants; however, adding nitrogen to irrigation water did not alleviate the reduced growth of these mutants. In addition to nitrogen status, levels of indole-3-acetic acid and gibberellin in five-week-old plants were also affected by the *aln* mutations. To test the possibility that ureides are remobilized from source to sink organs, we measured ureide levels in various organs. In wild-type plants, allantoate accumulated predominantly in inflorescence stems and siliques; this accumulation was augmented by disruption of its catabolism. Mutants lacking ureide transporters, ureide permeases 1 and 2 (UPS1 and UPS2), exhibited phenotypes similar to those of the ureide-degradation mutants, but had decreased allantoate levels in the reproductive organs. Transcript analysis in wild-type plants suggested that genes involved in allantoate synthesis and ureide transport were coordinately upregulated in senescing leaves.

**Conclusions:**

This study demonstrates that ureide degradation plays an important role in supporting healthy growth and development in non-legume Arabidopsis during and after transition from vegetative to reproductive stages.

**Electronic supplementary material:**

The online version of this article (10.1186/s12870-018-1491-2) contains supplementary material, which is available to authorized users.

## Background

Plants require nitrogen (N) in large quantities and its limited availability negatively impacts plant growth. Therefore, plants recycle and redistribute stored N in cellular macromolecules (i.e., proteins and nucleic acids) [1, 2]. Although chloroplast proteins, particularly ribulose-1,5-bisphosphate carboxylase/oxygenase, are the main sources of remobilized N [3–5], nucleic acids are also N sources [6, 7]. Purines and pyrimidines constitute the nitrogenous components of nucleic acids. The free forms of these nucleobases provide reusable N upon decomposition [8, 9]. Based on N content, purines are more valuable than pyrimidines, with four ring N atoms that are recycled eventually as ammonium.

Unless they are converted to nucleotides through salvage reactions, the free forms of purine nucleobases converge on xanthine, the first common intermediate of the purine catabolic pathway. In Arabidopsis [*Arabidopsis thaliana* (L.) Heynh.], xanthine is further degraded through a series of biochemical reactions that are mediated by seven enzymes distributed in the cytosol, peroxisomes, and endoplasmic reticulum (ER) [10, 11]. Xanthine is oxidized to urate by xanthine dehydrogenase (XDH) in the cytosol [12, 13]. Although there are two orthologous XDH proteins (AtXDH1 and AtXDH2), AtXDH1 is mostly responsible for XDH activities in Arabidopsis [14, 15]. Urate is then transported into the peroxisomes and converted to the ureide allantoin via three enzymatic reactions. The first reaction is mediated by urate oxidase, followed by the bifunctional enzyme allantoin synthase that catalyzes the last two reactions [16, 17]. Subsequent ureide degradation is accomplished stepwise by four ER-localized hydrolases, releasing four ammonium ions from one molecule of ureide. Allantoin is decyclized by allantoinase (ALN or allantoin amidohydrolase) to allantoate [18], which is degraded by allantoate amidohydrolase (AAH) to ureidoglycine and an ammonium ion [19]. Ureidoglycine is further hydrolyzed to release another ammonium ion by ureidoglycine aminohydrolase [20]. The resultant ureidoglycolate is finally metabolized to hydroxyglycine and then to glyoxylate and two ammonium ions, by ureidoglycolate amidohydrolase and subsequent non-enzymatic decomposition [21]. The purine-derived ammonium is likely re-assimilated for glutamine synthesis in the cytosol, as is that derived from proteolysis and subsequent amino acid deamination, although little is known about how this inorganic ion is exported from the ER. Although the purine-derived ureides allantoin and allantoate are the principal stores of fixed N_2_ in leguminous species of tropical and sub-tropical origin [22], the precise contribution of N derived from purines to general nitrogen metabolism remains largely unexplored in non-leguminous plants such as Arabidopsis [10].

Purine-catabolic mutants of Arabidopsis have been isolated, such as those lacking or with reduced expression of *XDH*, *ALN*, or *AAH*, and thus cannot use purine-derived N because of the block in ureide synthesis or degradation. A previous study in our laboratory demonstrated that simultaneous post-transcriptional gene silencing (PTGS) of *AtXDH1* and *AtXDH2* caused growth impairments and early senescence [23]. Similarly, other groups observed that PTGS and a T-DNA insertion mutation of *AtXDH1* caused early senescence [24, 25]. It was proposed that the ureides allantoin and allantoate function as antioxidant agents, and the phenotypes of *xdh* mutants were attributed to over-oxidation of living cells [24]. On the other hand, the effect of impaired ureide degradation on Arabidopsis growth has not been well described. Earlier studies reported that T-DNA insertion mutants of *ALN* and *AAH* (*aln* and *aah*) did not show any obvious morphological phenotype compared to wild type (WT) plants during early growth on agar medium plates containing sufficient inorganic N [18, 19, 26]. However, N recycling and remobilization are vigorously activated during and after the transition from vegetative to reproductive stages [2]. Thus, to investigate the physiological importance of ureide-derived N in Arabidopsis, the effects of defects in ureide degradation should be evaluated at later developmental stages. Our recent studies revealed that allantoin accumulation resulted in increased levels of abscisic acid (ABA) and jasmonic acid (JA) and enhanced stress hormone responses in young Arabidopsis seedlings [27, 28]. It is not known whether altered hormone levels persist and cause long-term effects on the subsequent growth and development of the ureide-degradation mutants.

Members of the ureide permease (UPS) family are regarded as ureide transporters. Certain members of the UPS family, such as PvUPS1 from French bean (*Phaseolus vulgaris* L.) and GmUPS1–1 and GmUPS1–2 from soybean (*Glycine max* L.), have been shown to mediate symplasmic transport and xylem loading of allantoin in nodulated roots [29–31]. PvUPS1 is possibly also involved in phloem loading of allantoin [32]. Although no evidence has so far been presented for long-distance translocation of the ureides in Arabidopsis, its genome contains five paralogous genes encoding UPS (AtUPS1–5). Among these paralogs, experiments involving heterologous expression in yeast and *Xenopus* oocytes have demonstrated that AtUPS1, AtUPS2, and AtUPS5 transport allantoin [33–35]. However, AtUPS1 and AtUPS2 exhibited several-fold higher affinity for uracil (AtUPS1 and AtUPS2) over allantoin, suggesting that their primary function *in planta* is providing the substrate to the uracil salvage pathway rather than transporting ureides [34, 35].

In this study, we conducted a detailed evaluation of the growth phenotype of Arabidopsis mutants defective in ureide degradation (*aln* and *aah*). We also conducted experiments to understand the underlying mechanism of growth attenuation of ureide-degrading mutants. Our results show that ureide degradation is important for the growth and development of Arabidopsis during and after the transition from vegetative to reproductive growth under long-day conditions. We also found N use efficiency (NUE) and endogenous phytohormone levels are affected in ureide-degradation mutants.

## Results

### Disruption of ureide degradation accelerates the transition to reproductive growth under long-day conditions

**Fig. 1:**
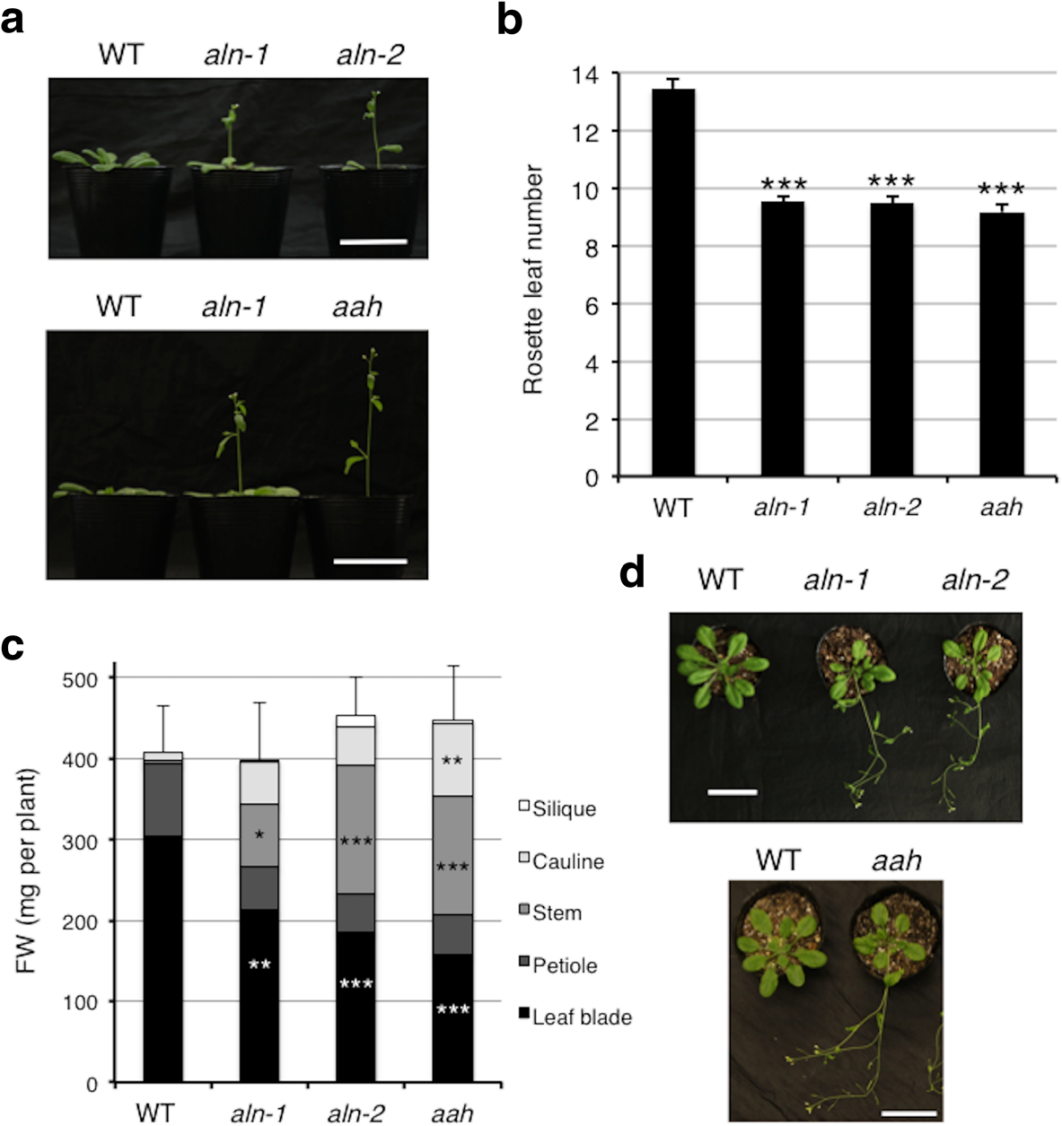
Early-flowering phenotype of *aln* and *aah* mutants. WT, *aln-1*, *aln-2*, and *aah* seeds were sown on soil and grown under long-day conditions. **a** Growth of representative plants for each genotype at 4 WAG. **b** Number of rosette leaves when the primary inflorescence stems were about 1 cm long (*n* ≥ 32). **c** Fresh weight (FW) of rosette leaves, petioles, stems, cauline leaves, and siliques from plants at 5 WAG (*n* ≥ 9). **d** Growth of representative plants for each genotype at 5 WAG. Bar = 5 cm. A linear model with the genotype as a factor was fitted to the data. Asterisks denote significant differences from WT (*P < 0.05, **P < 0.01, ***P < 0.001, two-tailed *t*-tests based on the model)

The *aln-1* and *aln-2* mutants carry loss-of-function alleles of *ALN* and are incapable of degrading allantoin; the *aah* mutant is deficient in allantoate hydrolysis due to the disruption of *AAH*. For more detailed information about the *aln-1*, *aln-2* and *aah* mutants, see Supporting Information Figs S1 and Figure S3 in Watanabe et al. [27]. In T-DNA insertion mutant lines in general, there could be mutations in addition to the T-DNA-tagged mutations of interest [36]. For this reason, we cannot exclude the possibility that mutant phenotypes that are only observed with a single mutant allele are due to inadvertent mutations. Therefore, subsequently we only consider the mutant phenotypes consistent between two independent mutant alleles of the *ALN* gene as true *aln* mutant phenotypes. In our laboratory conditions, consistent with previous reports [18, 19, 26], seedlings of *aln-1*, *aln-2* and *aah* mutants grown on half-strength Murashige and Skoog (1/2 MS) standard medium containing either gellan gum or agar did not show obvious morphological phenotypes before bolting [27, 28]. Although these mutants produced flowers as reported by Yang and Han [18], we noticed that *aah* plants bolted earlier than WT plants when plants were grown on 1/2 MS medium plates for 3 weeks (Additional file 1: Figure S1). To further test for early flowering phenotypes, we cultivated *aln-1*, *aln-2* and *aah* mutants in soil for a longer time. Consistently, *aah* plants as well as both *aln* (*aln-1* and *aln-2*) mutants started bolting and flowering as early as 4 weeks after germination (WAG), whereas WT plants remained non-bolting (Fig. 1a), suggesting the early flowering phenotype shown in Additional file 1: Figure S1 is not attributable to the growth conditions. The number of rosette leaves when the primary inflorescence stems emerged was about 14 in WT, but fewer than 10 in ureide-degradation mutants (Fig. 1b). The three mutants also displayed slight but significant decreases in foliar chlorophyll (Additional file 2: Figure S2). At 5 WAG, although the mutants and WT had similar fresh weights (FW) of the entire aerial parts, the mutants had less FW of rosette leaf blades and more FW of the stems and cauline leaves compared to wild type (Fig. 1c, d). Leaf blades and petioles of the ureide-degradation mutants were significantly shorter than WT (Additional file 3: Table S1). Together, these phenotypes indicated that disruption of ureide degradation accelerated the transition from vegetative to reproductive growth under long-day conditions.

### Disruption of ureide degradation impairs reproductive growth

**Fig. 2:**
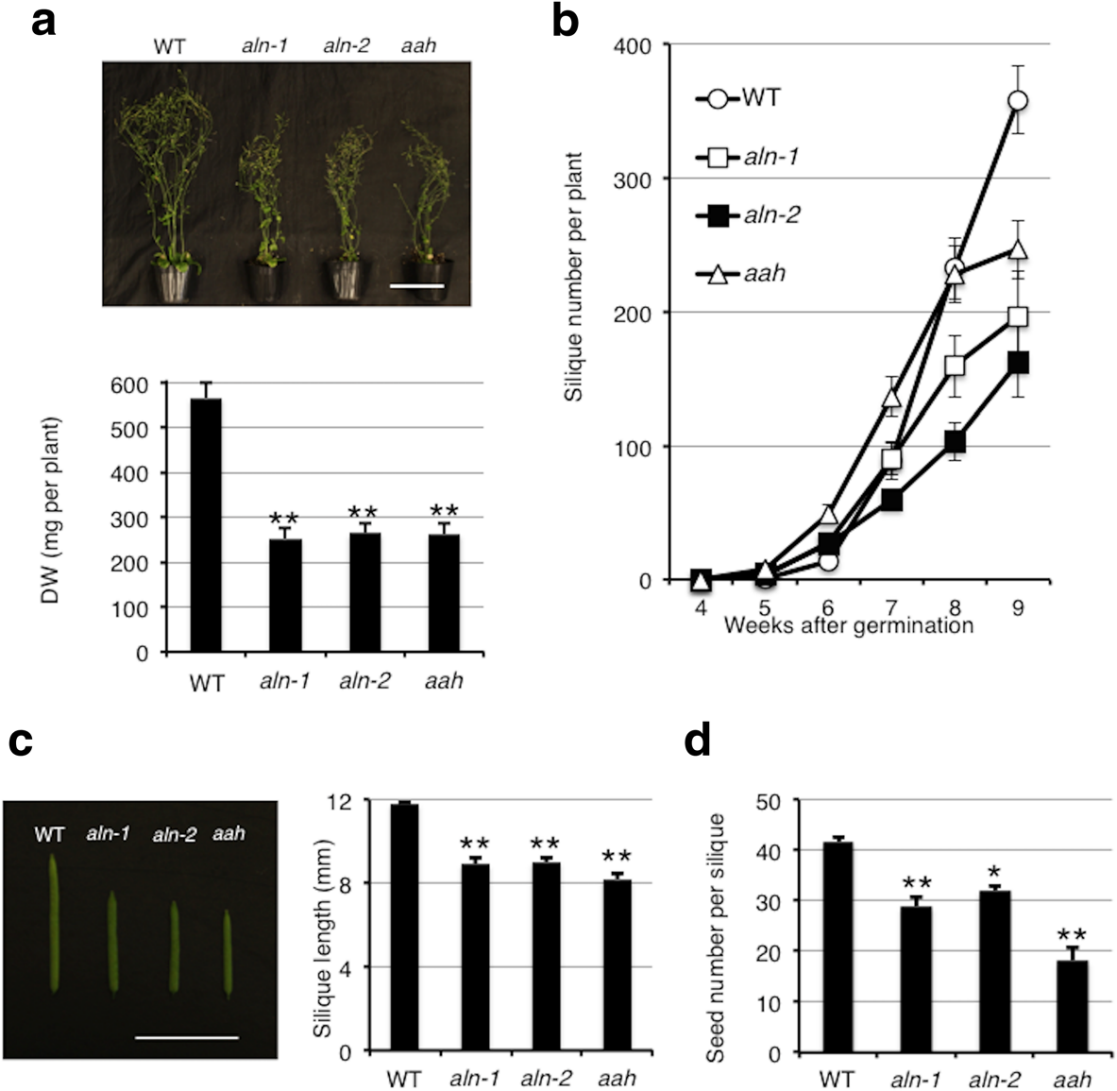
Reduced growth and fertility of *aln* and *aah* mutants. **a** Growth of representative plants and dry weight (DW) for each genotype at 9 WAG (*n* ≥ 15). Bar = 10 cm. **b** The number of siliques produced per plant from 4 to 9 WAG (*n* ≥ 5). **c** The length of siliques at 9 WAG (*n* = 10). Bar = 1 cm. **d** The number of seeds in a silique from plants at 9 WAG (*n* = 6). A linear model with the genotype as a factor was fitted to the data. Asterisks denote significant differences from WT (*P < 0.01, **P < 0.001, two-tailed *t*-tests based on the model)

Since purine catabolism is a putative N-recycling metabolism and early flowering is a typical N-deficient symptom, we hypothesized that ureide degradation substantially contributes to efficient N use, especially in later growth stages. To test this idea, we examined the *aln* and *aah* mutant phenotypes at the late growth stages. At 9 WAG, the three mutants were smaller than the WT, with whole-plant dry weights (DW) approximately half that of WT plants (Fig. 2a). Due to the early-flowering phenotype, the mutants (particularly the *aah* mutants) tended to form more siliques than did WT plants during the early stages of inflorescence development (up to 7 WAG; Fig. 2b). However, consistent with the lower DW, total silique numbers of the three mutants were reduced to 50 to 67% of WT at 9 WAG (Fig. 2b). Moreover, the siliques of these mutants were significantly shorter and contained fewer seeds than those of WT plants (Fig. 2c, d). These reproduction-associated phenotypes demonstrated that ureide degradation plays important roles in the late stages of Arabidopsis growth.

### Disruption of ureide degradation accelerates leaf senescence in mature plants

**Fig. 3:**
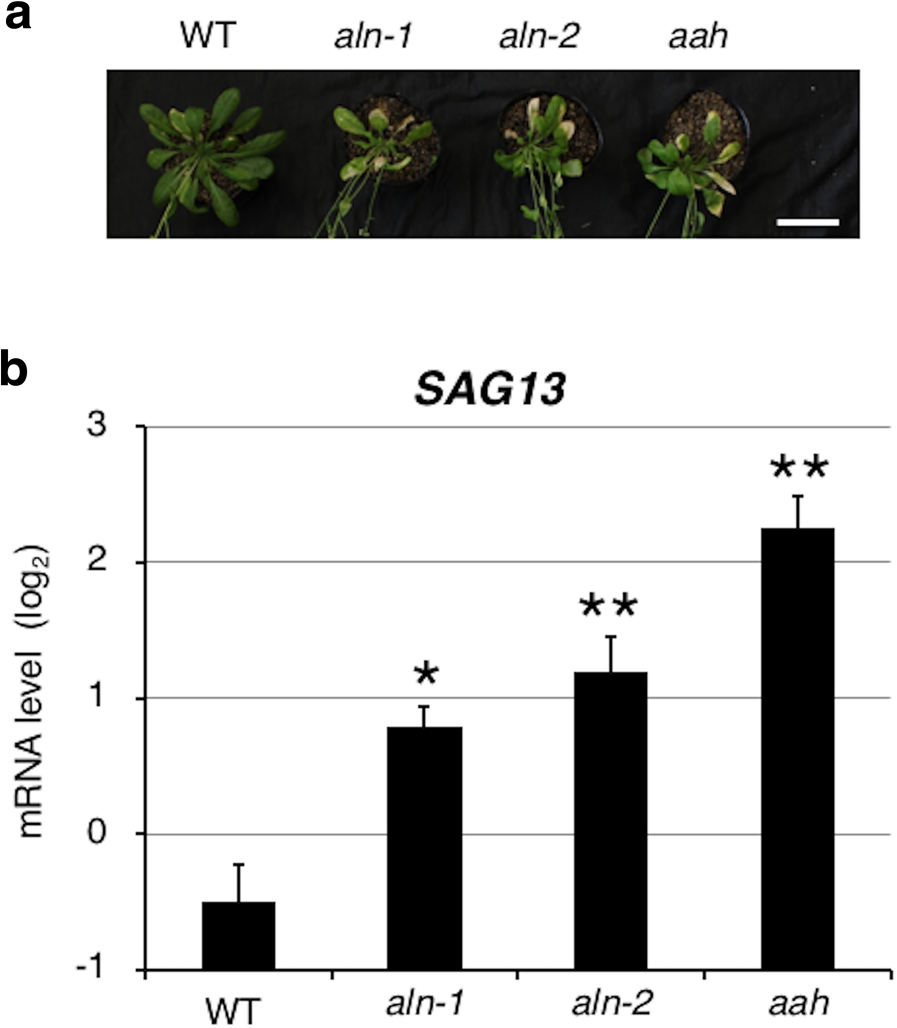
Accelerated leaf senescence of *aln* and *aah* mutants. **a** Growth of representative plants for each genotype at 9 WAG. Leaves from each genotype are arranged in order of decreasing age from left to right. Bar = 5 cm. **b** *SAG13* expression levels. RNA was extracted from all leaves from plants at 7 WAG. Relative mRNA levels were determined using *ACT2*, *UBC9* and *MON1* as reference genes and presented as the log_2_ of the mean and standard error of three independent biological replicates. A linear model with the genotype as a factor was fitted to the data. Asterisks denote significant differences from WT (*P < 0.01, **P < 0.001, two-tailed *t*-tests based on the model)

The vegetative-to-reproductive transition generally coincides with the onset of leaf senescence. We therefore examined whether ureide degradation mutants showed accelerated senescence. At 9 WAG, the *aln* and *aah* mutants showed more yellowing of the rosette leaves than did WT plants (Fig. 3a & Additional file 4: Figure S3). To test the early senescence phenotype in these mutants at the molecular level, we analyzed the expression of a canonical senescence marker, *SENESCENCE-ASSOCIATED GENE 13* (*SAG13*), in rosette leaves from 7-week-old plants by quantitative reverse transcription-PCR (qRT-PCR) (Fig. 3b). Consistent with their early senescence symptoms, the mutants showed significantly higher levels of *SAG13* expression compared with the WT. These findings indicated that defective ureide degradation leads to precocious leaf senescence in mature Arabidopsis plants.

### Disruption of ureide degradation affects N status but exogenous N supply did not restore growth of *aln* mutants

**Fig. 4:**
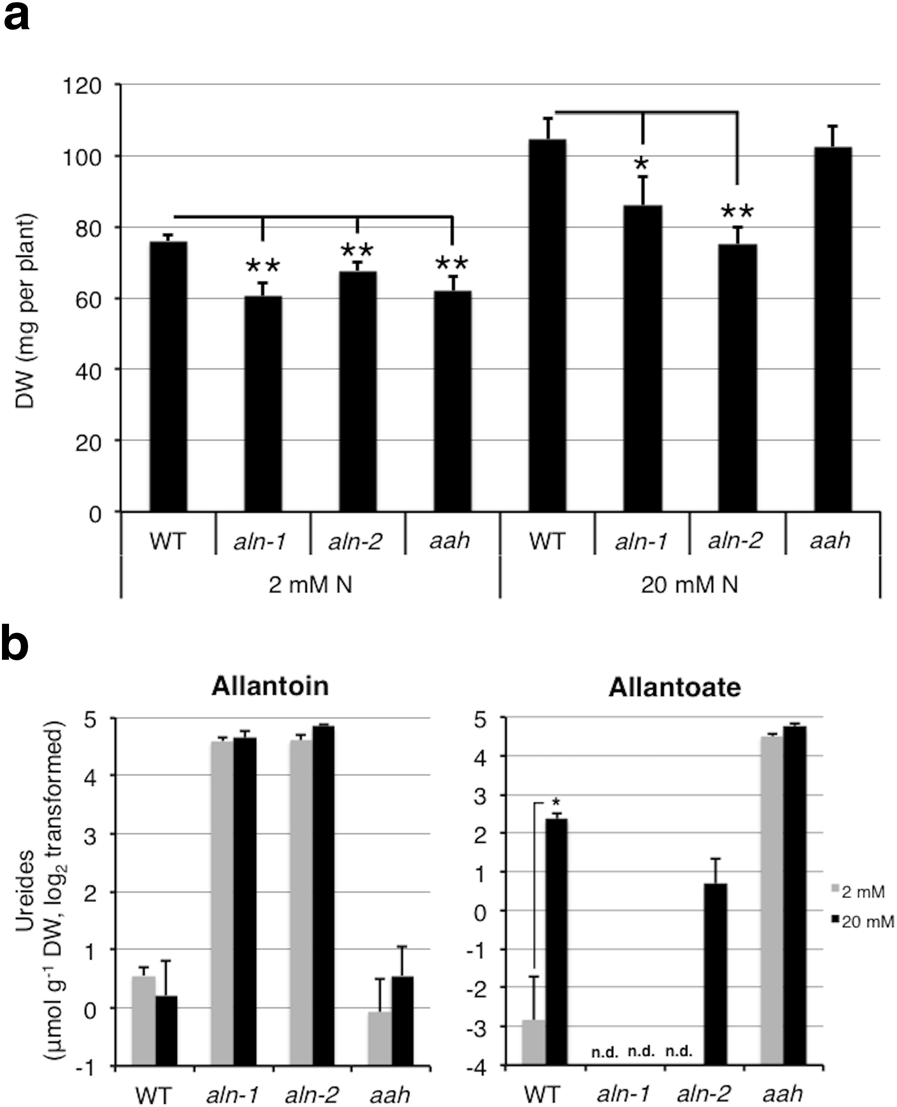
Effect of nitrogen (N) deficiency on the growth of *aln* and *aah* mutants. Aseptically grown 2 WAG seedlings were transplanted to pots containing vermiculite and perlite, and then further grown for 4 weeks with weekly irrigation of N-deficient (2 mM N) and sufficient (20 mM N) nutrient solution. **a** Dry weight (DW) of whole shoots (*n* = 16). A linear model with the genotype as a factor was fitted to the data. Asterisks denote significant differences between WT and mutant plants (*P < 0.05, **P < 0.01, two-tailed *t*-tests based on the model). **b** Allantoin and allantoate levels were determined on a dry weight (DW) basis for shoots (*n* = 3). Asterisks denote significant differences in allantoin or allantoate contents between N-deficient and -sufficient conditions for each genotype (*P < 0.05, two-tailed *t*-tests). n.d., not detected

Since *aln* and *aah* mutants displayed N-deficiency-like phenotypes as they grew toward maturity (Figs 1, 2, 3), we examined relative N content, NUE, and carbon-to-nitrogen (C/N) ratio as indicators of N status in these mutants. At 9 WAG, all three mutants exhibited significant differences in these parameters relative to WT, with increases in relative N content but decreases in NUE and C/N ratio (Table 1). The results suggested that *aln* and *aah* mutants were relatively inefficient at using N for carbon assimilation and dry matter production. On the other hand, the higher relative N content in ureide-degradation mutants suggested that N uptake was not affected. To further investigate whether ureides provide substantial N nutrients, we next tested the effect of N shortage on plant growth. We hypothesized that the mutants would exhibit growth reduction relative to WT earlier in low-N than N-sufficient conditions, because N recycling and remobilization processes are activated earlier in low-N conditions to compensate for limited N supply. Two-week-old aseptically grown seedlings were carefully removed from the medium to avoid damaging the roots, transplanted to a mixture of vermiculite and perlite, and then grown for an additional 4 weeks under N-sufficient (20 mM) or low-N (2 mM) conditions. It is worth noting that plants grown under these conditions bolted and matured earlier than those under the soil growth conditions described in Fig. 1, 2, 3. At 6WAG, both WT and mutants were apparently at the reproductive stage under these growth conditions, regardless of the N regime; they had already bolted and developed siliques (Additional file 5: Figure S4). The *aah* mutants showed DW comparable to WT under N-sufficient conditions, and significantly lower DW than WT under low-N conditions (Fig. 4a). Contrary to our expectation, however, the *aln-1* and *aln-2* mutants showed significantly decreased DW compared to WT under both N-sufficient and deficient conditions. Irrespective of N regimes, *aln* and *aah* mutants accumulated high levels of allantoin and allantoate, respectively (Fig. 4b), suggesting that N regimes do not affect ureide biosynthesis. However, WT plants grown under low N showed significantly decreased allantoate levels, implying that degradation of allantoate is activated by N deficiency. A small amount of allantoate was detected in the *aln-2* plants under N-sufficient conditions, probably due to the spontaneous hydrolysis of allantoin [37].

**Table 1:** Nitrogen (N) content, nitrogen use efficiency (NUE), and carbon:nitrogen ratio (C/N) in 9-week-old *aln* and *aah* mutants

N-status	Genotype			
Parameter	Wild type	*aln-1*	*aln-2*	*aah*
N (% dry weight)	3.39 ± 0.13	4.45 ± 0.13**	4.10 ± 0.04*	4.09 ± 0.15*
NUE	29.65 ± 1.14	22.53 ± 0.65**	24.38 ± 0.26**	24.55 ± 0.89**
C/N	11.60 ± 0.57	8.60 ± 0.27**	9.23 ± 0.22**	8.89 ± 0.38**

Values are means ± standard error (*n* = 4)

Significant differences using two-tailed *t*-tests based on the linear model at: *, *P* < 0.01 and **, *P* < 0.001

### Disruption of allantoin degradation changed phytohormone balance at 5 WAG

We previously reported that young seedlings of *aln* mutants showed significantly enhanced drought and osmotic stress tolerance, along with moderately elevated levels of ABA and JA [27, 28]. Besides controlling stress responses, these hormones affect the levels and activity of other hormones, as well as plant growth and development. Therefore, the impaired growth of the ureide-degradation mutants could be due to altered hormone levels rather than inefficient N utilization. To test this idea, we determined ABA, JA, jasmonoyl-L-isoleucine (JA-Ile), indole-3-acetic acid (IAA), gibelellin (GA_4_), salicylic acid (SA), *trans*-zeatin (tZ), N^6^-(∆^2^-isopentenyl)adenine (iP) levels in aboveground parts of soil-grown *aln* and *aah* mutants at 5 WAG (Table 2). Although the mutant plants had a higher proportion of reproductive organs than WT at 5 WAG (Fig. 1c, d), we thought that this time point was the best to estimate the effect of stress hormones on the growth reduction, because it was just before the differences in plant sizes between WT and mutants became visible. Compared to the WT, the *aln-1* and *aln-2* mutants had significantly increased levels of IAA and GA_4_. In ABA and JA-Ile levels, the *aln-2* mutant showed statistically significant differences relative to WT. However, we consider that we do not have significant differences in these hormones in *aln* plants, since we could not detect them in both alleles. In *aah* mutants, none of the hormones showed significant changes relative to the WT levels.

**Table 2:** Phytohormone levels in 5-week-old *aln* and *aah* mutants

Phytohormone	Genotype			
	WT	*aln-1*	*aln-2*	*aah*
ABA (ng g^− 1^ DW)	78.6 ± 3.2 b	90.8 ± 6.4 ab	113.2 ± 3.3 a	86.2 ± 8.1 b
JA (ng g^− 1^ DW)	99.5 ± 60.8 a	86.0 ± 26.9 a	188.0 ± 71.1 a	132.5 ± 57.9 a
JA-Ile (ng g^− 1^ DW)	5.4 ± 2.3 b	6.6 ± 1.6 ab	13.9 ± 1.0 a	6.9 ± 1.6 ab
IAA (ng g^− 1^ DW)	180.7 ± 2.4 c	402.8 ± 72.3 b	718.3 ± 64.5 a	264.5 ± 40.2 bc
GA4 (ng g^− 1^ DW)	2.3 ± 0.5 c	4.6 ± 0.5 ab	5.4 ± 0.2 a	3.4 ± 0.2 bc
tZ (ng g^− 1^ DW)	10.8 ± 0.5 a	7.6 ± 1.1 a	6.4 ± 1.1 a	7.8 ± 1.3 a
iP (ng g^− 1^ DW)	1.6 ± 0.1 a	2.6 ± 0.4 a	2.2 ± 0.3 a	1.7 ± 0.4 a
SA (mg g^− 1^ DW)	3.2 ± 0.1 a	3.9 ± 1.1 a	2.9 ± 0.2 a	4.0 ± 0.3 a

Values are means ± standard error (*n* = 3)

Different letters indicate significant differences determined by Tukey’s HSD test (*P* < 0.05)

### Reproductive tissues contain relatively high allantoate levels that are augmented by disruption of AAH

**Fig. 5:**
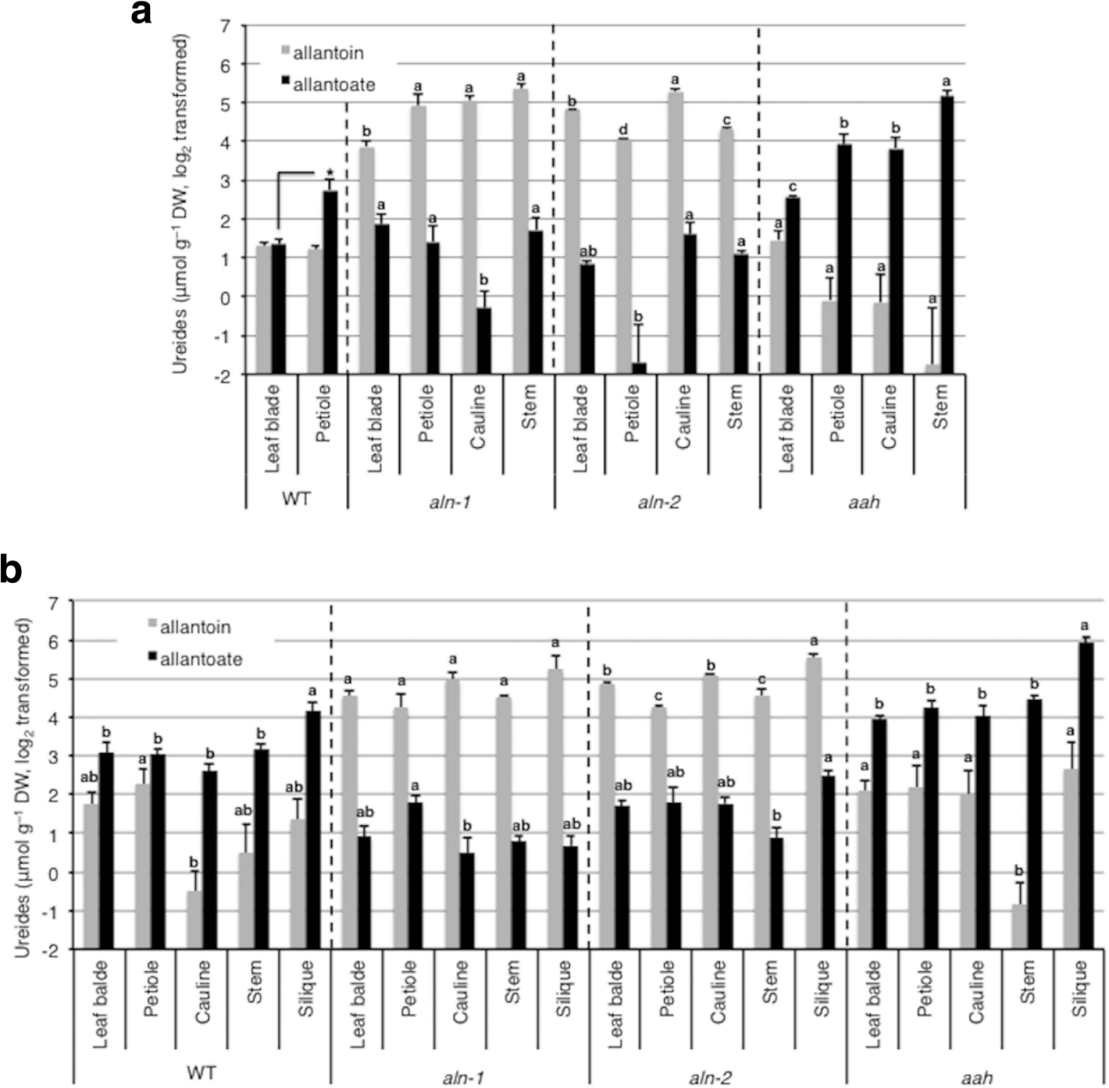
Ureide concentrations in the aboveground tissue and seeds of *aln* and *aah* mutants. **a** Five WAG plants were cut into leaf blades, petiole, cauline leaves, and inflorescence stems, and allantoin and allantoate levels were determined on a dry weight (DW) basis. Asterisk denotes significant differences of allantoate contents between leaf blade and petiole in 5-week-old WT (*n* = 3; *P < 0.05, two-tailed *t*-test). Different letters indicate significant differences among allantoin or allantoate levels in each mutant line (*n* = 3; *P* < 0.05, Tukey’s HSD test). **b** Nine WAG plants were cut into leaf blades, petioles, cauline leaves, inflorescence stems, and siliques, and allantoin and allantoate levels were determined on a DW basis. Different letters indicate significant differences among allantoin or allantoate levels in each genotype (*n* = 3; *P* < 0.05, Tukey’s HSD test)

To investigate the possibility that ureides are utilized as recycled N, we next examined the tissue distribution of allantoin and allantoate in the aboveground parts of Arabidopsis plants, because source-to-sink allocation and redistribution of N constitute important physiological processes for efficient N use when plants grow to maturity and produce seeds, or when available N is limited. At 5 WAG, when WT plants had not bolted or had very small inflorescence stems (Fig. 1c, d), the rosette leaves contained equal levels of allantoin in the leaf blades and petioles and 3-fold higher levels of allantoate in the petioles (Fig. 5a). By this stage, both *aln* and *aah* mutants had already started bolting (Fig. 1) and had significantly higher levels of allantoin and allantoate, respectively, than the WT, with allantoate concentrated in inflorescence stems and allantoin distributed more evenly (Fig. 5a). At 9 WAG, WT plants exhibited higher levels of allantoate than allantoin in every tissue examined (leaf blades, petioles, cauline leaves, stems and siliques), and the highest level was detected in siliques (Fig. 5b). Such accumulation profiles were consistent and augmented in the *aah* mutant, in which allantoate degradation is blocked. Compared to allantoate, allantoin was distributed relatively evenly in the WT and the *aah* mutant, although the *aln* mutants appeared to accumulate this ureide slightly more in siliques and cauline leaves than in the other tissues. Possibly, this evenly distributed pattern of allantoin was due to the saturation of this ureide. At both 5 and 9 WAG, a small amount of allantoate was detected from the two alleles of *aln* mutant plants, probably due to the spontaneous hydrolysis of allantoin [37]. In contrast to other plant parts, mature dry seeds from the WT contained very little allantoate compared with allantoin. Even in the *aah* mutant seeds, allantoate levels were lower than allantoin (Additional file 6: Figure S5). On the other hand, seed allantoin levels were comparable with, or even higher than those in other plant parts. The seed ureide contents indicate that allantoate in siliques was not localized to seeds, and this ureide does not function as N storage in Arabidopsis seeds.

### Mutations of UPS1 and UPS2 caused growth defects similar to ureide degradation dysfunction but reduced ureide levels in reproductive tissues

**Fig. 6:**
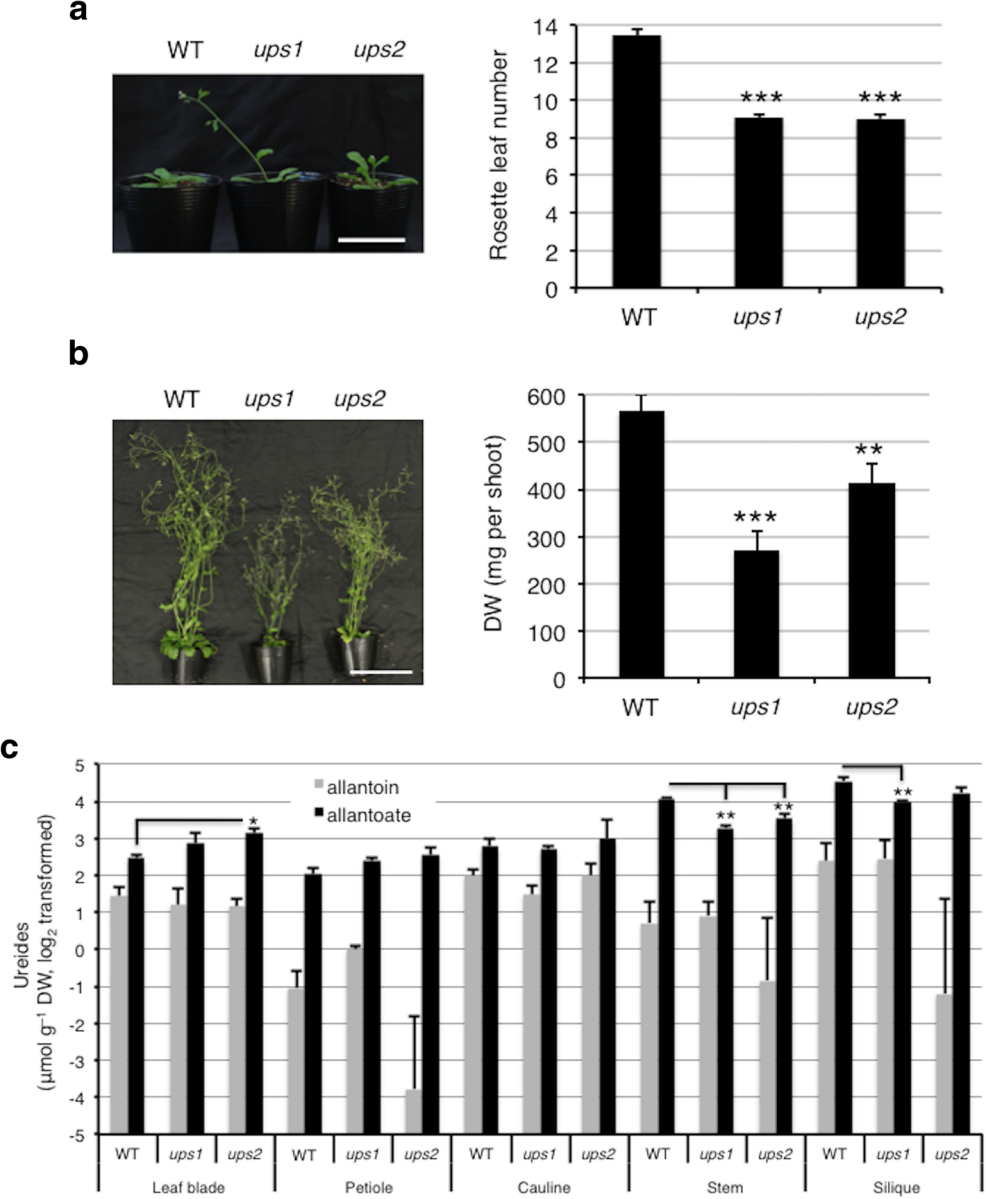
Growth phenotype and ureide accumulation in the *ups1* and *ups2* mutants. **a** Early flowering of *ups1* and *ups2* mutants. Representative plants for each genotype are shown at 4 WAG. Bar = 5 cm. Number of rosette leaves when the primary inflorescence stems were about 1 cm long (*n* ≥ 28). **b** Growth of representative plants and dry weight (DW) for each genotype at 9 WAG (*n* ≥ 12). Bar = 10 cm. **c** Ureide concentrations in aboveground tissue. Seven WAG plants were cut into leaf blades, petioles, cauline leaves, inflorescence stems, and siliques, and allantoin and allantoate levels were determined on a DW basis (*n* = 3). A linear model with the genotype as a factor was fitted to the data. Asterisks denote significant differences between WT and mutant plants (*P < 0.05, **P < 0.01, ***P < 0.001, two-tailed *t*-tests based on the model)

The above results suggested that not only the degradation, but also the tissue partitioning of ureides, which involves membrane transport, is critical for supporting growth at the later stages of Arabidopsis development. Previously, it was shown that impaired N remobilization due to a block in source–sink transport of amino acids negatively affected seed production [38]. We therefore investigated the effect of disrupting ureide transport on plant growth and the tissue distribution of allantoin and allantoate using *ups1* and *ups2*, two knockout mutants for the Arabidopsis UPS proteins, AtUPS1 and AtUPS2, respectively (Additional file 7: Figure S6). Schmidt et al. [34] previously reported that PTGS of *UPS1* and T-DNA insertion of *UPS2* did not affect plant growth either on agar plates or soil. However, similar to *aln* and *aah* mutants, *ups1* and *ups2* mutants showed early flowering (Fig. 6a), and at 9 WAG under long-day conditions, displayed a reduction in whole-plant DW of 50% and 25%, respectively, compared with the WT (Fig. 6b). The discrepancy between the present and the previous study might be due to differences in growth conditions and/or observation timing, since detailed methodological information was not available in Schmidt et al. [34].

The tissue distribution of the two ureides was determined at 7 WAG when the WT and the two *ups* mutants were bolting and developing siliques (Additional file 8: Figure S7). AtUPS1 and AtUPS2 have been reported to transport allantoin but exhibit little affinity for allantoate in heterologous assay systems [33, 34]. However, no significant difference was found in allantoin levels between the WT and the mutants in any of the tissues examined (Fig. 6c). To our surprise, compared to the WT, allantoate levels were significantly lower in inflorescence stems of the *ups1* and *ups2* mutants and in siliques of the *ups1* mutant, but increased in rosette leaf blades of the *ups2* mutant. These results showed that, like disruption of ureide degradation, dysfunction of UPS transporters negatively affected the reproductive growth of Arabidopsis. However, the effects on the distribution of the two ureides varied and, notably, allantoate levels decreased in the reproductive tissues of the *ups* mutants.

### Genes involved in ureide synthesis and transport are activated in source tissues at the vegetative-to-reproductive phase transition

**Fig. 7:**
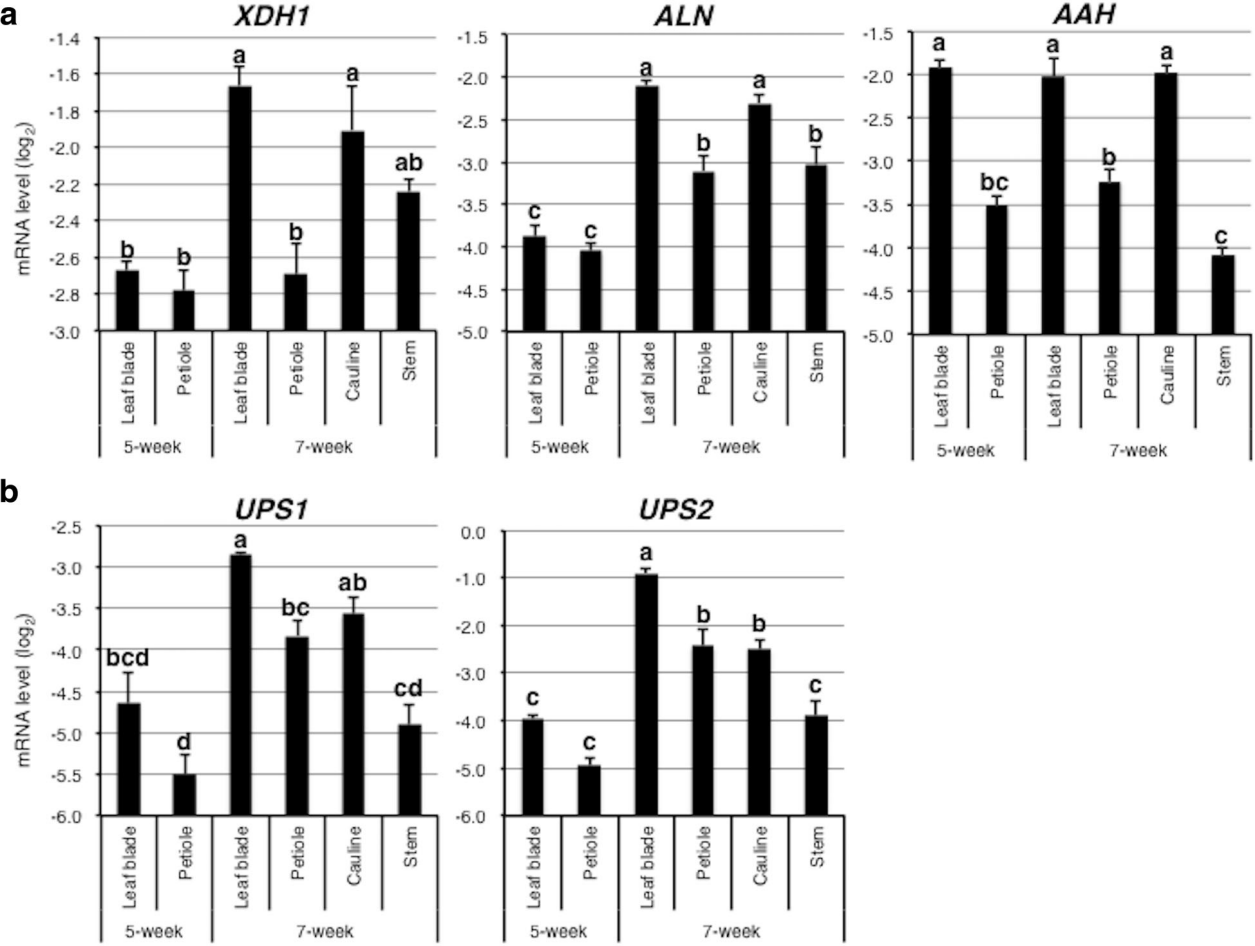
Changes in mRNA levels of genes involved in ureide synthesis, degradation, and possible transport during leaf senescence. **a** Expression of ureide synthesis (*XDH1*) and degradation (*ALN* and *AAH*) genes. **b** Expression of ureide transport genes (*AtUPS1* and *AtUPS2*). RNA was extracted separately from leaf blades and petioles from 5 and 7 WAG plants, and cauline leaves, inflorescence stems, and siliques from 7 WAG plants. Relative mRNA levels were determined using *ACT2*, *UBC9*, and *MON1* as a reference, and presented as the log_2_ of the mean and standard error of three independent biological replicates for each gene. Different letters indicate significant differences determined by Tukey’s HSD test (*P* < 0.05)

To examine gene expression changes associated with tissue distribution of allantoin and allantoate, we determined changes in transcript levels of genes involved in ureide metabolism (*XDH1*, *ALN*, and *AAH*) and transport (*AtUPS1* and *AtUPS2*) in WT plants upon transition from the vegetative (5 WAG, see Fig. 1d) to the reproductive phase (7 WAG; see Additional file 8: Figure S7). qRT-PCR showed that in rosette leaf blades during the growth phase transition, transcript levels for *XDH1* and *ALN* increased, while those for *AAH* remained constant (Fig. 7a). Consistent with our data, *XDH1* and *ALN* are expressed at relatively high levels in senescent leaves according to publicly available microarray data [39]. In the reproductive organs, the *XDH1*, *ALN* and *AAH* genes showed higher expression in cauline leaves than in stems; however, stems at 7 WAG showed higher transcript levels of *ALN* than rosette leaf blades and petioles at 5 WAG. These results suggest that ureide synthesis, particularly of allantoate, is activated in both source (rosette and cauline leaves) and sink (inflorescence stems) organs. Coincident with the increase in *XDH1* and *ALN* expression, *AtUPS1* and *AtUPS2* transcript levels increased in leaf blades and petioles (Fig. 7b). These expression patterns are also consistent with publicly available microarray data; these *UPS* genes were highly expressed in senescent leaves [39]. In the reproductive organs, *AtUPS1* and *AtUPS2* genes showed higher expression in cauline leaves than in stems. These results suggest that UPS-mediated ureide partitioning is activated in source organs as Arabidopsis plants transition from vegetative to reproductive growth and the subsequent formation of seeds.

## Discussion

Degradation of the ureides allantoin and allantoate is crucial for utilization of symbiotically fixed N in ureide-type legumes such as nodulated soybean [40]. In plants other than ureide-type legumes, it has long been regarded as an N-recycling metabolism; however, there is little evidence that ureide degradation is important for growth and development [10]. Seedlings of Arabidopsis mutants with defective ureide degradation (catalyzed by ALN and AAH) indeed grow normally on medium containing sufficient inorganic N [18, 19, 26]. In this study, we examined the phenotypes of *aln-1*, *aln-2* and *aah* mutants during and after the transition from vegetative to reproductive growth. When plants were grown long-term under long-day conditions, the three ureide-degradation mutants flowered earlier than the wild type (Fig. 1), and had significant reductions in biomass and seed production (Fig. 2). Moreover, these mutants displayed accelerated leaf senescence (Fig. 3), although allantoin and allantoate have been suggested to function as antioxidant agents [24]. Nourimand and Todd [41] showed that another *aln* T-DNA insertion line (*aln-3*) grown on soil under short-day conditions (8 h light and 16 dark) exhibited comparable growth with WT plants. Because short-day conditions suppress the transition to reproductive stages, the growth phenotypes we observed may be specific for long-day conditions.

### Possible reasons for growth reduction: Inefficient N use and altered stress responses

At least two possibilities might explain the impaired growth of the ureide-degrading mutants after transition to reproductive growth. The first is that these mutants might experience N deficiency as they grow to maturity, due to blocked recycling of ureide-derived N. This possibility is supported by the following observations: (i) the mutants displayed symptoms typical of N-starvation (Figs 1, 2, 3); (ii) these symptoms were accompanied by unfavorable N status, as indicated by decreased NUE and C:N ratio (Table 1). The possibility that ureides are a source of recycled N is also supported by the observation that WT plants exposed to low-N conditions had significantly reduced allantoate levels (more than 24-fold lower than in plants grown under N-sufficient conditions; Fig. 4b). It is plausible that this metabolic response reflects the accelerated degradation of allantoate to liberate ammonium for re-assimilation, since Arabidopsis has been shown to utilize allantoin as an N nutritional source during the early seedling stage [18, 19, 33]. However, growth of *aln* mutants was not restored to WT levels by sufficient N supply (Fig. 4a), thus the physiological significance of recycled N derived from ureides is still questionable and we cannot exclude other possibilities as the cause of growth attenuation.

The second possibility to explain the impaired growth of the ureide-degrading mutants is the antagonistic relationship between plant growth and stress responses [42]. We previously found that allantoin overaccumulation enhances the levels of stress hormones, ABA and JA, in the aseptically grown 2-week-old seedlings, thereby providing better stress protection at this growth stage [27, 28]. Despite ABA and JA overaccumulation, the aseptically grown *aln* mutants did not show obvious morphological phenotypes at 2 WAG [27, 28]. In this study, therefore, we tested phytohormone levels at 5 WAG to investigate if ABA and JA could be factors in growth reduction in ureide-degradation mutants. At 5 WAG, the *aln-2* but not *aln-1* mutant showed statistically significant differences in ABA and JA-Ile levels (Table 2). It is still difficult to conclude whether ABA and JA affected the growth reduction in the mutants; however, those stress hormones are unlikely to be a main cause of the growth reduction, since neither *aln-1* nor *aah* mutants showed significant differences in ABA and JA levels compared with WT plants (Table 2). It also seems that ABA and JA had a minor impact on the early onset of flowering, since those hormones are known to suppress the transduction from vegetative to reproductive stages [43, 44]. Both *aln* mutants showed significantly increased GA_4_ and IAA levels. We cannot distinguish if the differences in phytohormone levels are due to the different developmental stages between WT and mutants (Fig. 1c, d). Nevertheless, these hormones might have affected the early transition to the reproductive stage, since GA_4_ promotes flowering and auxin positively regulates GA signaling and biosynthesis [45, 43]. It is also possible that overaccumulation of IAA affected the antagonistic interaction between auxin and cytokinin and restricted shoot growth [46]. However, these altered hormone levels are unlikely to be the sole cause of growth reduction in the ureide-degradation mutants, because the *aah* mutant did not show significant changes in any phytohormones (Table 2).

There is also a possibility that the phenotypes of ureide-degradation mutants, including altered phytohormone balance, are due to a toxic effect of ureide overaccumulation on the plant cellular functions. Overaccumulation of the ureide precursor uric acid resulted in a severe defect in peroxisome function [15]. While uric acid accumulates in peroxisomes, ureides are localized in the ER. Ureides overacculation might negatively affect ER function in the *aln* and *aah* mutants.

### Contribution of ureides to N remobilization and stress protection

To further explore the importance of ureide utilization in Arabidopsis growth, we also investigated the distribution patterns of ureides. In the aboveground parts, WT plants accumulated substantially more allantoate in influorescence stems and siliques after the onset of reproductive development (Figs 5a, b, 6c). Gene expression data of WT plants are consistent with this ureide accumulation pattern; the upstream genes of allantoin *XDH1* and *ALN* were upregulated during leaf senescence, while *AAH* remained unchaged (Fig. 7a). In French bean, similarly, allantoate levels were significantly increased in stems after the onset of the reproductive development [47]. This allantoate in French bean was thought to originate from senescent leaves but not nodules, because N-fixation activity was significantly decreased after the onset of reproductive development. Allantoate is the first intermediate substrate to release ammonium in the purine-catabolic pathway, thus it is possible that this acyclic ureide is the preferred form for temporary storage and transport during senescence-induced N remobilization in both leguminous and non-leguminous plants. In this study, since rosette and cauline leaves of Arabisopsis showed higher *AAH* expression than petioles and stems (Fig. 7a), it appears that Arabisopsis plants quickly degraded allantoate and subsequently released ammonium in leaves. Presumably, the released ammonium was reassimilated into amino acids and translocated to upper parts of the plants. However, allantoate itself seemed to be translocated from source to sink organs, since this ureide tended to accumulate in repoductive organs of not only WT but also the *aah* mutant. To our knowledge, the only reported plant proteins that transport allantoate are GmUPS1–1 and GmUPS1–2, two UPS homologs in soybean [30]. Thus, it would be interesting to investigate whether Arabidopsis translocates allantoate via specific transporters. Contrary to other plant parts, seeds contained significantly higher levels of allantoin than allantoate even in *aah* mutants (Fig. 5c). Allantoin, albeit being one of the two major ureides, responds to a variety of stresses in several species (see [27] and references therein), including Arabidopsis, where it probably plays roles in stress protection as recently reported in the *aln* mutants [27, 28, 41, 48, 49]. Taking into account its suggested roles in scavenging reactive oxygen species and activating stress responses [24, 27, 28, 50, 51], allantoin might contribute to stress protection during the maturation, dormancy, and germination of seeds, as well as early growth of seedlings. In support of this view, seed allantoin levels in fifteen rice cultivars are highly and positively correlated with seedling survival at low temperatures and under drought conditions [52].

Although they were demonstrated to primarily transport nucleobases such as uracil [34, 35], AtUPS1 and AtUPS2 are still the most likely ureide transporters reported to date in non-N_2_-fixing species [53]. Two *ups* mutants decreased allantoate but not allantoin contents in sink tissues (inflorescence stems and siliques). Since allantoin but not allantoate is a substrate of those transporters [33, 34], we think that mutation of *UPS* genes attenuated allantoin transport from source to sink organs, and allantoin in both source and sink organs was rapidly converted to allantoate. This idea is supported by the gene expression profile of WT plants at 7 WAG (Figs 7). At this stage, compared with 5 WAG, *ALN* expression levels were relatively higher in both source and sink organs, while *UPS1* and *UPS2* expression levels were higher only in source but not in sink organs.

We hypothesized that ureide transport duing N remobilization through AtUPS1 and AtUPS2 is important for efficient N use and that the *ups1* and *ups2* T-DNA insertion mutants would phenocopy, at least partly, the *aln* and *aah* mutants as they grew to maturity. The early flowering and reduced growth phenotypes of the two *ups* mutants supported this prediction (Fig. 6a, b), suggesting that they experienced a perturbation in ureide-derived N usage. It is unlikely that growth phenotypes of *ups* mutants during and after onset of reproductive stages are the consequence of attenuated uracil transport, because the importance of uracil salvage declines after germination when de novo synthesis takes over [34].

If ureides are a substantial source of remobilized N, it is interesting to estimate how much N is recycled and remobilized by ureide degradation. According to data from previous studies, the levels of glutamine and asparagine, two representatives of remobilized N, were roughly 40 and 10 μmol g^− 1^ DW in 6-week-old WT leaves, and 20 and 10 μmol g^− 1^ DW in siliques, respectively, in Arabidopsis [38, 54]. Since these amido-amino acids contain two atoms of N per molecule, those correspond to approximately 80 and 20 μmol g^− 1^ N in 6 WAG leaves and 40 and 20 μmol g^− 1^ N in siliques, respectively, on a dry matter basis. On the other hand, ureide levels in this study were 8.3 μmol g^− 1^ DW (allantoin 2.8 and allantoate 5.5 μmol g^− 1^ DW) in 7 WAG WT leaves and 29.4 μmol g^− 1^ DW (allantoin 5.8 and allantoate 23.6 μmol g^− 1^ DW) in siliques (Fig. 6c). Since each ureide has four atoms of N, they correspond to 33.2 and 117.6 μmol g^− 1^ N in the leaves and siliques, respectively, on a dry matter basis. Given the comparison between amido-amino acids and ureides, it is possible that ureides constitute a substantial percentage of remobilized N in Arabidopsis.

## Conclusions

Our detailed phenotypic analysis demonstrated that, under long-day conditions, ureide degradation contributes to healthy growth and development during and after transition to the reproductive stage. Mutations blocking ureide degradation affected efficient N utilization and phytohormone balances, illustrating the physiological importance of this catabolic pathway. Ureides appear to be utilized as remobilized N, since they accumulated more in sink organs such as stems and siliques. Consistent with the phenotypes of ureide-degradation mutants, the impairment of ureide transporters resulted in growth attenuation. Our gene expression analysis using WT plants suggested that ureide degradation and transport are coordinately regulated followed by the progression of senescence.

## Materials and methods

### Plant materials and standard growth conditions

The *Arabidopsis thaliana* (L.) Heynh. Columbia-0 accession was used as WT. Seeds of the following mutants and T-DNA insertion lines were obtained from the Arabidopsis Biological Resource Center (Ohio State University, Columbus, Ohio, USA): *aah* (SALK_112631) [19, 27], *aln-1* (SALK_000325; formerly called *aln*) [18, 27], *aln-2* (SALK_146783) [27], *ups1* (SAIL_549_F06; this study), and *ups2* (SALK_044551C; also known as *ups2–2*) [34]. For aseptic growth, seeds were surface-sterilized and cold-treated at 4 °C for 2 days then placed on half-strength Murashige and Skoog medium containing 1% (*w*/*v*) sucrose and solidified with 0.3% (w/v) gellan gum. Seeds were germinated and grown at 23 °C under long-day light conditions (16-h light, 8-h darkness) with a light intensity of 70 μmol photons m^− 2^ s^− 1^. For growth in soil pots, imbibed seeds were sown on garden soil (Hanasaki Monogatari, Akimoto Tensanbutsu, Iga, Japan) which contains the following nutrients: 290 mg L^− 1^ N; 620 mg L^− 1^ phosphorus; 285 mg L^− 1^ potassium; and small amounts of magnesium, boron, iron, and manganese. Plants were irrigated with tap water as needed to prevent the soil from drying out and supplemented weekly with 1:2000-diluted HYPONeX 6–10-5 fertilizer [neat liquid containing 6% (w/v) N, 10% (w/v) phosphoric acid, 5% (w/v) water-soluble potassium and micronutrients; HYPONeX Japan Corp., Ltd., Osaka, Japan).

### Growth phenotype analysis

FW was measured for the aerial parts of 5-week-old plants that were separated into rosette leaf blades, petioles, cauline leaves, inflorescence stems, and siliques. For DW measurements, whole shoots of 9 WAG plants were dried in a forced-air oven at 65 °C. Since it was difficult to collect intact roots, they were not used for any analyses. Flowering time was defined as the number of rosette leaves when primary inflorescences were 1 cm in length. For silique formation, the number of siliques on both the main inflorescence stem and the side branches was counted every week from 4 to 9 WAG. The silique length and seed numbers per silique were evaluated from 10 independent samples where one sample contained 10 representative fully expanded mature siliques selected visually from a single 9-week-old plant. The length of siliques was measured using ImageJ software version 1.45 (http://rsbweb.nih.gov/ij/).

### Ureide content

The ureides allantoin and allantoate were determined by modified methods of Todd and Polacco [18]. Powdered freeze-dried samples were ground in 40 (for WT, *ups1* and *ups2* mutants) or 100 vol (for *aln-1*, *aln-2* and *aah* mutants) 50 mM potassium phosphate buffer (pH 7.0) using a mortar and pestle. Homogenates were transferred to 1.5 mL microtubes and centrifuged at 18,000×*g* for 20 min at 4 °C. Supernatants were collected in 1.5 mL microtubes and centrifuged again at 18,000×*g* for 5 min at 4 °C to further spin down residues. Supernatants were collected, and allantoin and allantoate were quantified after chemical transformation to glyoxylate. To transform allantoin to glyoxylate, 10 μL of supernatants were mixed with 20 μL distilled water and 10 μL 0.5 M NaOH, followed by heating at 100 °C for 8 min. After cooling on ice for 5 min, samples were mixed with 10 μL of 0.65 M HCl, heated at 100 °C for 4 min, and cooled on ice for 5 min. To transform allantoate to glyoxylate, 10 μL of supernatants were mixed with 30 μL distilled water and 10 μL 0.15 M HCl, and heated at 100 °C for 4 min. To transform ureidoglycolate to glyoxylate, 10 μL of supernatants were mixed with 10 μL 0.5 M NaOH, and placed at room temperature for 1 min. After the chemical transformation to glyoxylate, sample volume and pH were adjusted with 0.4 M Na_2_HPO_4_-KH_2_PO_4_ buffer (pH 7.0), and 10 μL of phenylhydrazine (10 mg in 3 mL of water) were added. After 5 min at room temperature, samples were placed on ice, and mixed with 50 μl of ice-cold concentrated HCl and 10 μL of potassium ferricyanide (50 mg in 3 mL of water). Samples were placed at room temperature at least for 15 min, followed by centrifugation at 15,000×*g* for 10 min. Absorbance of supernatants was measured using a spectrophotometer at 535 nm. Allantoin and allantoate concentrations were determined by the subtraction of levels of glyoxylate converted from allantoate and ureidoglycolate, respectively.

### Phytohormone quantification

We used plants at 5 WAG in spite of different developmental stages between WT and mutant plants, since this time seemed just before mutants started showing differences in biomass production. We used whole shoots, but not individual plant parts, since wounding severely affect hormone biosynthesis, especially JA. In addition to wounding effect, stems and cauline leaves in WT plants were too small to collect enough weight for hormone measurements; some WT plants even had not started bolting at 5 WAG. Therefore, in this study, whole aerial parts of 5-week-old plants were freeze-dried and ground to a fine powder. Subsequently, phytohormones were extracted and quantified following the methods of Tsukahara et al. [55] with minor modifications. Samples were mixed with 4 mL of 80% (v/v) acetonitrile containing 1% (v/v) acetic acid and known amounts of stable isotope-labeled internal standards at 4 °C for 1 h. After removal of cell debris by centrifugation at 3000×*g* for 10 min, the resultant residues were again extracted with 80% (v/v) acetonitrile containing 1% (v/v) acetic acid. The two supernatants were combined, evaporated in a vacuum centrifugal evaporator (miVac quattro concentrator, Genevac Ltd., Ipswich, UK) and dissolved in 1% (v/v) acetic acid. The extracts were loaded onto a pre-equilibrated Oasis HLB column cartridge (Waters Corporation, Milford, MA, USA). After washing with water acidified with 1% acetic acid, the column was eluted with 80% (v/v) acetonitrile containing 1% (v/v) acetic acid. The eluted samples were evaporated to obtain extracts in water acidified with 1% acetic acid, and loaded onto a pre-equilibrated Oasis MCX column cartridge (Waters). The cartridge was washed with 1% acetic acid (v/v), and the acidic fraction was eluted with 80% acetonitrile containing 1% acetic acid. A portion of the acidic eluate was used for SA quantification. The cartridge was further washed with 5% aqueous ammonia, and the basic fraction was eluted with 40% acetonitrile containing 5% ammonia and used for tZ and iP quantification. The remaining acidic fraction was evaporated, dissolved in 1% acetic acid, and loaded onto pre-equilibrated Oasis WAX column cartridges (Waters). The cartridge was washed with 1% acetic acid (v/v) and 80% acetonitrile, and remaining hormones were eluted with 80% acetonitrile containing 1% acetic acid. This acidic eluate was used for IAA, GA_4_, ABA, JA, and JA-Ile quantification. All fractions were analyzed on an Agilent 6410 Triple Quadrupole system with a ZORBAX Eclipse XDB-C18 column and MassHunter software version B.01.02 (Agilent Technology, Palo Alto, CA, USA).

### Elemental analysis and NUE

Aerial parts of soil-grown, 9 WAG plants were freeze-dried and ground into fine powder, and N and C concentrations were measured by combustion and thermal conductivity separation in a CHN analyzer (Perkin Elmer 2400 Series II CHNS/O Analyzer, Perkin Elmer, Norwalk, Connecticut, USA). NUE was calculated following equation of Steenbjerg and Jakobsen [56].

$$ \mathrm{NUE}=\mathrm{Sw}\div {\mathrm{N}}_{\mathrm{total}} $$

where *Sw* is shoot dry weight and *N*_*total*_ is total N content in the shoot.

### N-deficient conditions

Aseptically grown, 2 WAG seedlings were transplanted into 16-cell trays (28 cm × 28 cm × 5 cm; each seedling planted in one cell) containing a mixture of vermiculite and perlite (3:2 [*v*/v]), and grown for a further 4 weeks, irrigated weekly with culture solution containing either 2 or 20 mM N (1000 mL per tray; Additional file 9: Table S2). Aerial parts were harvested for DW and ureide measurements.

### Gene expression analysis

qRT-PCR was performed according to the SYBR Green method outlined in Watanabe et al. [27]. Total RNA was extracted using the Nucleospin RNAII extraction kit (Macherey-Nagel GmbH & Co, Düren, Germany), and cDNA was synthesized from 500 ng of RNA using ReverTra Ace qPCR RT kit (Toyobo, Osaka, Japan). Subsequently, mRNA levels were quantified by qRT-PCR using the KAPA SYBR FAST qPCR Kit (Kapa Biosystems, Inc., Woburn, MA, USA) in a 7300 Real-Time PCR System (Applied Biosystems, Foster City, CA, USA). The PCR program consisted of an initial denaturation at 95 °C for 10 min, followed by 40 cycles of 95 °C for 15 s and 60 °C for 1 min. The specificity of primers was confirmed using dissociation curve analysis. The relative log_2_-expression values were obtained by subtracting the *C*_*t*_ values for each target gene from the mean of *C*_*t*_ value of reference genes, *ACTIN 2* (*ACT2*), *UBIQUITIN CONJUGATING ENZYME 9* (*UBC9*), and *MONENSIN SENSITIVITY 1* (*MON1*). Primer sequences for target genes are listed in Additional file 10: Table S3.

### Statistical analysis

Quantitative measurements were repeated at least three times and data were expressed as the mean ± standard error. For the gene expression levels and IAA and ureide contents, log_2_-transformed values were used to meet requirements for homogeneity of variance. Comparisons of two groups were performed with unpaired two-tailed *t-*tests using Microsoft Excel. Comparison of WT and two or more mutants were performed with two-tailed *t*-tests based on one-factor linear models in the R programming environment. For balanced data, comparison of three or more groups was performed with one-way ANOVA, followed by Tukey’s HSD tests in the R programming environment. For the statistical analysis of gene expression data (Fig. 7), plant age and parts were regarded as one factor because stems and cauline leaves were not included at 5 WAG. For unbalanced data, comparison of three or more groups was performed with Tukey-Kramer multiple comparison tests in the R programming environment.

## Additional files

Additional file 1: **Figure S1.** Three-week-old *aah* mutants grown on gellan gum medium. Two WAG seedlings grown on half-strength Murashige and Skoog medium containing 0.3% gellan gum were carefully removed to avoid damaging roots, transplanted to new medium and grown for an additional week. (PDF 1951 kb)

Additional file 2: **Figure S2.** Chlorophyll contents of rosette leaves from four WAG plants. Chlorophylls were measured by a hand-held optical sensor (SPAD-502Plus, Konica Minolta Sensing, Inc., Tokyo, Japan) and represented in SPAD units. Values are means ± standard error (*n* ≥ 15). Different letters indicate significant differences determined by Tukey-Kramer test (*P* < 0.05). (PDF 391 kb)

Additional file 3: **Table S1.** Leaf morphometrics of *aln* and *aah* mutants. (DOCX 26 kb)

Additional file 4: **Figure S3.** All leaves of representative plants for each genotype at 9 WAG. (PDF 7344 kb)

Additional file 5: **Figure S4.** Six WAG plants grown nitrogen-sufficient or -deficient conditions. Aseptically grown 2 WAG seedlings were transplanted to pots containing vermiculite and perlite, and then further grown for 4 weeks with weekly irrigation of N-deficient (2 mM N) or sufficient (20 mM N) nutrient solution. Bar = 5 cm. (PDF 2434 kb)

Additional file 6: **Figure S5.** Ureide concentrations in the dry seeds of *aln* and *aah* mutants. Asterisks denote significant differences between WT and mutant plants (*n* = 3; **P < 0.001, two-tailed *t*-tests). n.d., not detected. (PDF 1069 kb)

Additional file 7: **Figure S6.** Characterization of *ups1* and *ups2* mutants. (a) Diagram of the T-DNA insertion in *AtUPS1* in the *ups1* mutant. Arrows indicate PCR primers; white boxes indicate untranslated regions; black boxes indicate exons; black lines indicate introns. (b) PCR-based genotyping of the *ups1* mutant using primers specific to *AtUPS1* (F1 and R1) and the left border sequence of the T-DNA (SAIL LB1). (c) Diagram of the T-DNA insertion in *AtUPS2* in the *ups2* mutant. Arrows indicate PCR primers; white boxes indicate untranslated regions; black boxes indicate exons; black lines indicate introns. (d) PCR-based genotyping of the *ups1* mutant using primers specific to *AtUPS2* (F2 and R2) and the left border sequence of the T-DNA (LBa1). (e) Semi-quantitative reverse transcription-PCR for estimating *AtUPS1* and *AtUPS2* mRNA levels in the mutant lines. *VHA-A* expression was simultaneously analyzed as an internal control. (PDF 865 kb)

Additional file 8: **Figure S7.** Growth of representative *ups1* and *ups2* mutants. Plants were grown at 23 °C in soil for 7 weeks under long-day conditions. (PDF 257 kb)

Additional file 9: **Table S2.** Composition of liquid culture media with low (2 mM) and standard (20 mM) concentrations of inorganic nitrogen. (DOCX 28 kb)

Additional file 10: **Table S3.** Primers used in this study. (DOCX 32 kb)

## Abbreviations

AAH: Allantoate amidohydrolase
ABA: Abscisic acid
ACT2: Actin 2
ALN: Allantoinase
C: Carbon
DW: Dry weight
ER: Endoplasmic reticulum
FW: Fresh weight
GA_4_: Gibberellin
IAA: Indole-3-acetic acid
iP: *N*^6^-(∆^2^-isopentenyl)adenine
JA: Jasmonic acid
JA-Ile: Jasmonoyl-L-isoleucine
MON1: Monensin sensitivity 1
N: Nitrogen
NUE: Nitrogen use efficiency
PTGS: Post-transcriptional gene silencing
qRT-PCR: Quantitative reverse transcription-PCR
SA: Salicylic acid
SAG13: Senescence-associated gene 13
tZ: *Trans*-zeation
UBC9: Ubiquitin conjugating enzyme 9
UPS: Ureide permease
WAG: Weeks after germination
WT: Wild type
XDH: Xanthine dehydrogenase
