# Supplementary figures and images for: Disruption of ureide degradation affects plant growth and development during and after transition from vegetative to reproductive stages
Source: BMC Plant Biol. 2018 Nov 20;18:287. doi: 10.1186/s12870-018-1491-2 (PMC6245725; doi:10.1186/s12870-018-1491-2)

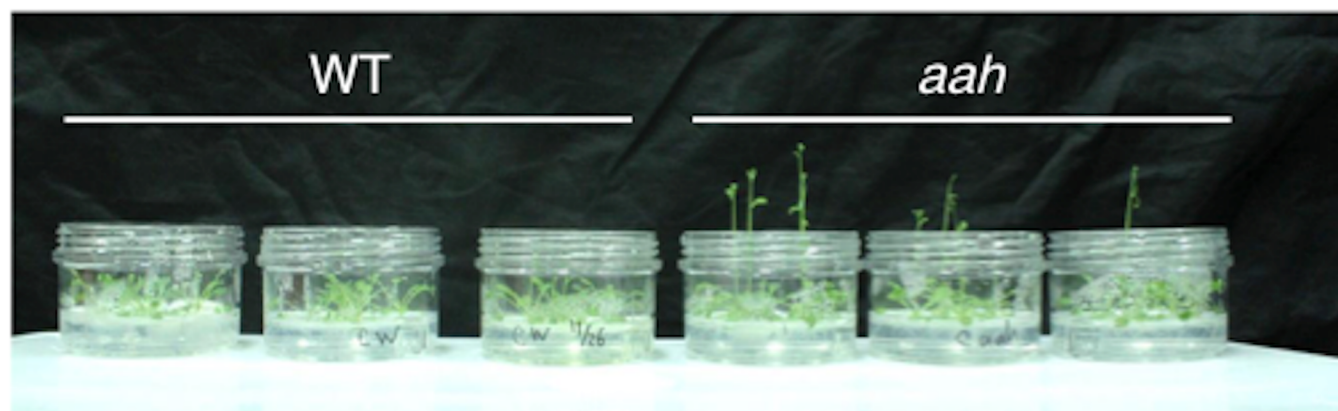

Supplement: Supplementary file 1 — Figure S1. Three-week-old aah mutants grown on gellan gum medium. Two WAG seedlings grown on half-strength Murashige and Skoog medium containing 0.3% gellan gum were carefully removed to avoid damaging roots, transplanted to new medium and grown for an additional week. (PDF 1951 kb) [file 12870_2018_1491_MOESM1_ESM.pdf]

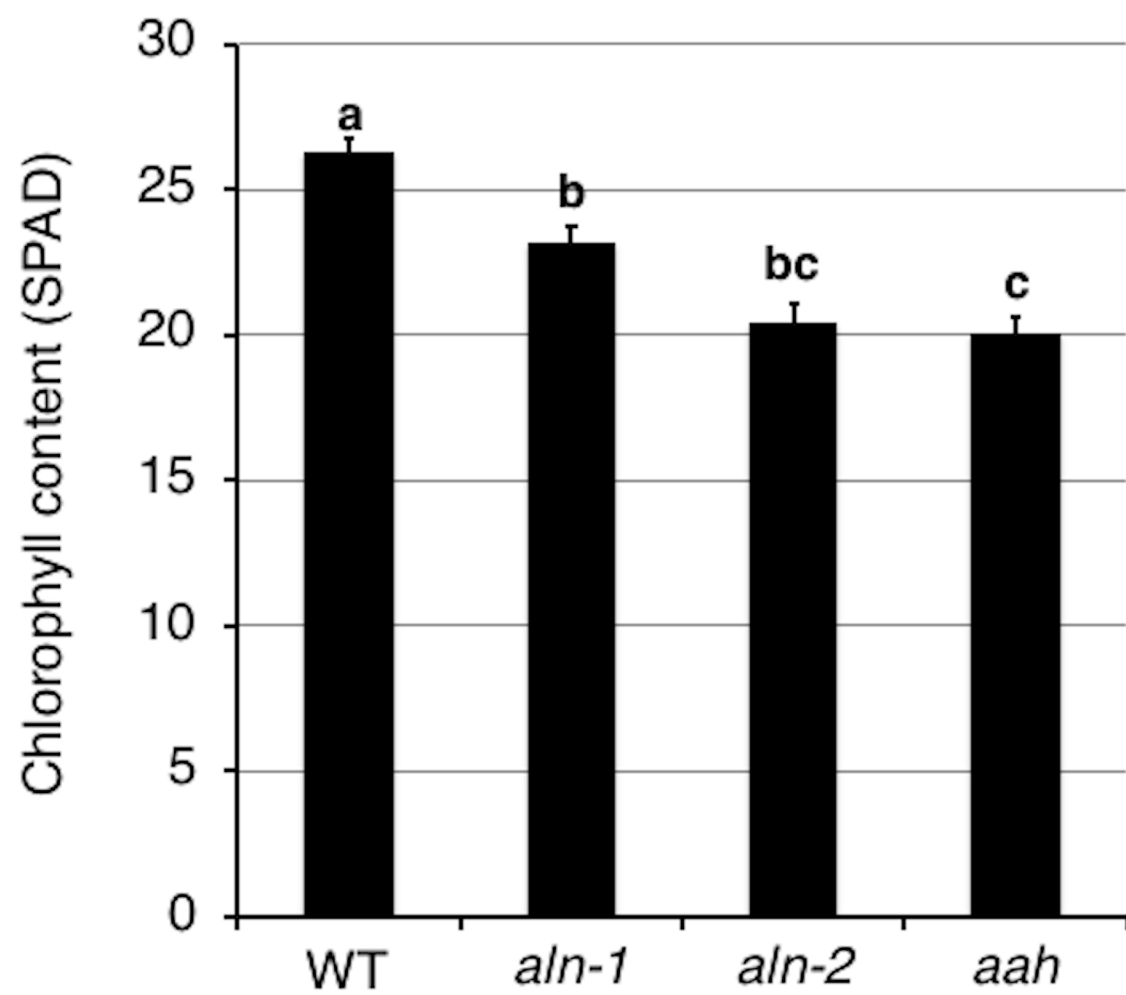

Supplement: Supplementary file 2 — Figure S2. Chlorophyll contents of rosette leaves from four WAG plants. Chlorophylls were measured by a hand-held optical sensor (SPAD-502Plus, Konica Minolta Sensing, Inc., Tokyo, Japan) and represented in SPAD units. Values are means ± standard error (n ≥ 15). Different letters indicate significant differences determined by Tukey-Kramer test (P < 0.05). (PDF 391 kb) [file 12870_2018_1491_MOESM2_ESM.pdf]

WT

*aln-1*

*aln-2*

*aah*

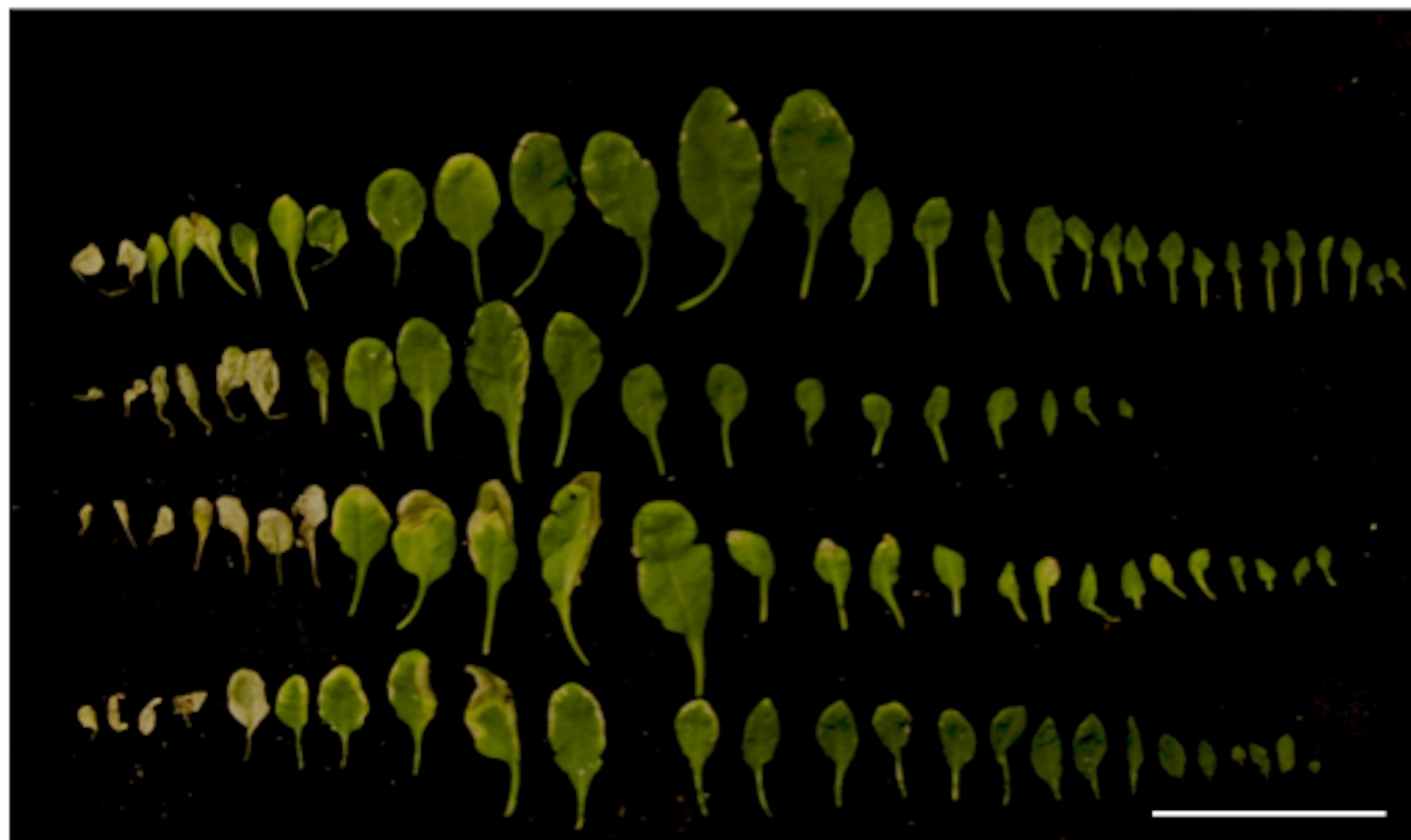

Supplement: Supplementary file 4 — Figure S3. All leaves of representative plants for each genotype at 9 WAG. (PDF 7344 kb) [file 12870_2018_1491_MOESM4_ESM.pdf]

WT    *aln-1*    *aln-2*    *aah*

2 mM

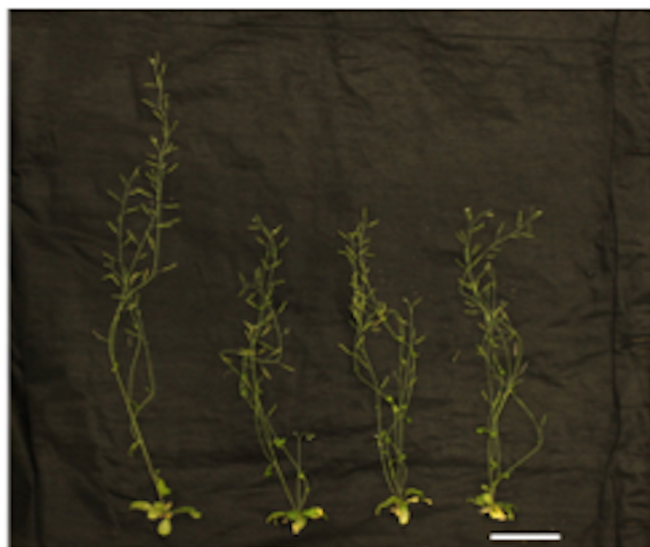

WT    *aln-1*    *aln-2*    *aah*

20 mM

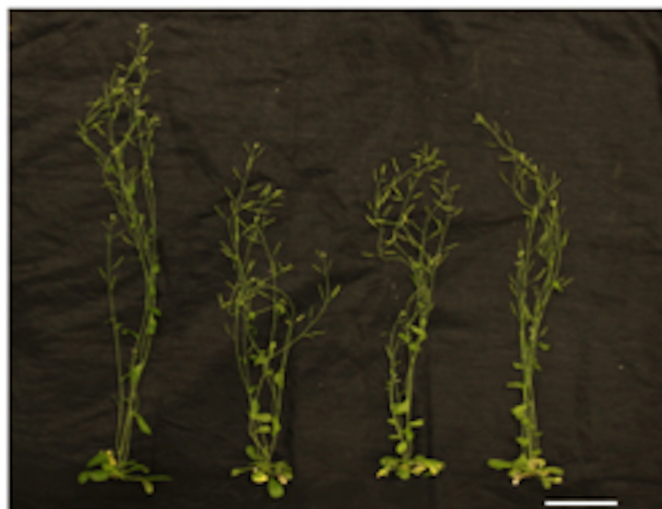

Supplement: Supplementary file 5 — Figure S4. Six WAG plants grown nitrogen-sufficient or -deficient conditions. Aseptically grown 2 WAG seedlings were transplanted to pots containing vermiculite and perlite, and then further grown for 4 weeks with weekly irrigation of N-deficient (2 mM N) or sufficient (20 mM N) nutrient solution. Bar = 5 cm. (PDF 2434 kb) [file 12870_2018_1491_MOESM5_ESM.pdf]

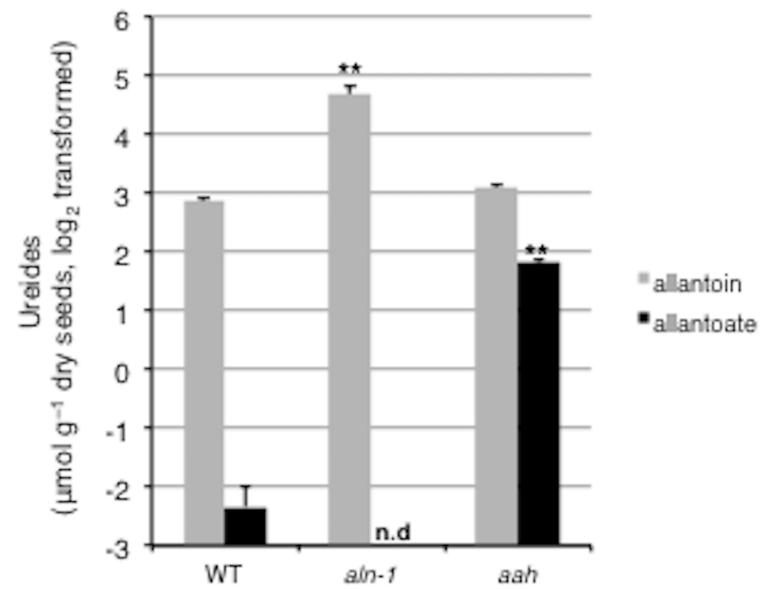

Supplement: Supplementary file 6 — Figure S5. Ureide concentrations in the dry seeds of aln and aah mutants. Asterisks denote significant differences between WT and mutant plants (n = 3; **P < 0.001, two-tailed t-tests). n.d., not detected. (PDF 1069 kb) [file 12870_2018_1491_MOESM6_ESM.pdf]

**a**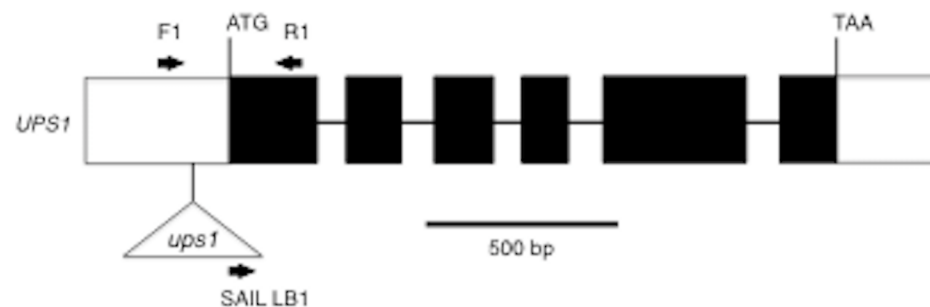**b**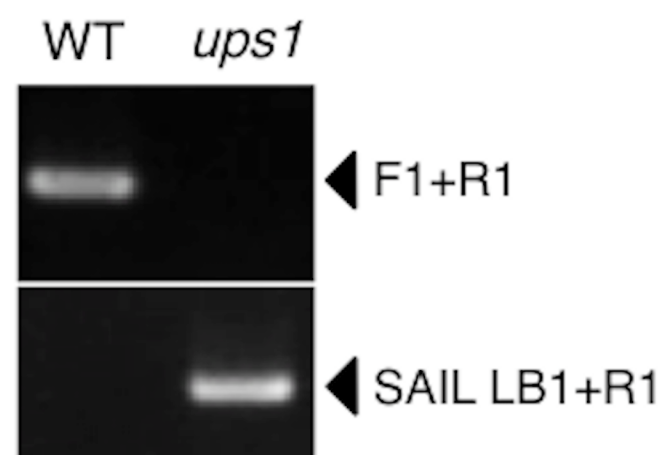**c**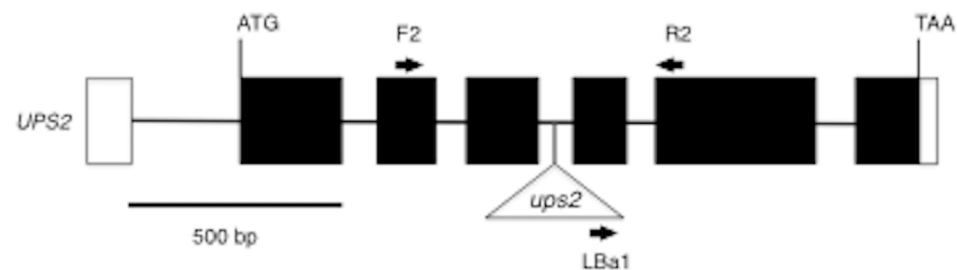**d**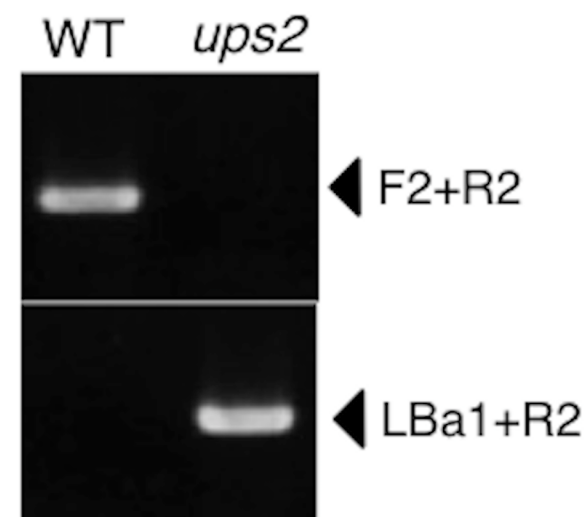**e**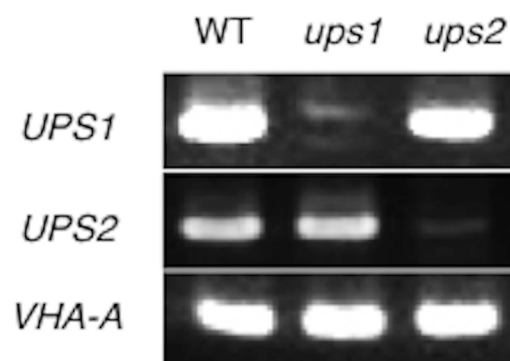

Supplement: Supplementary file 7 — Figure S6. Characterization of ups1 and ups2 mutants. (a) Diagram of the T-DNA insertion in AtUPS1 in the ups1 mutant. Arrows indicate PCR primers; white boxes indicate untranslated regions; black boxes indicate exons; black lines indicate introns. (b) PCR-based genotyping of the ups1 mutant using primers specific to AtUPS1 (F1 and R1) and the left border sequence of the T-DNA (SAIL LB1). (c) Diagram of the T-DNA insertion in AtUPS2 in the ups2 mutant. Arrows indicate PCR primers; white boxes indicate untranslated regions; black boxes indicate exons; black lines indicate introns. (d) PCR-based genotyping of the ups1 mutant using primers specific to AtUPS2 (F2 and R2) and the left border sequence of the T-DNA (LBa1). (e) Semi-quantitative reverse transcription-PCR for estimating AtUPS1 and AtUPS2 mRNA levels in the mutant lines. VHA-A expression was simultaneously analyzed as an internal control. (PDF 865 kb) [file 12870_2018_1491_MOESM7_ESM.pdf]

WT

*ups1*

*ups2*

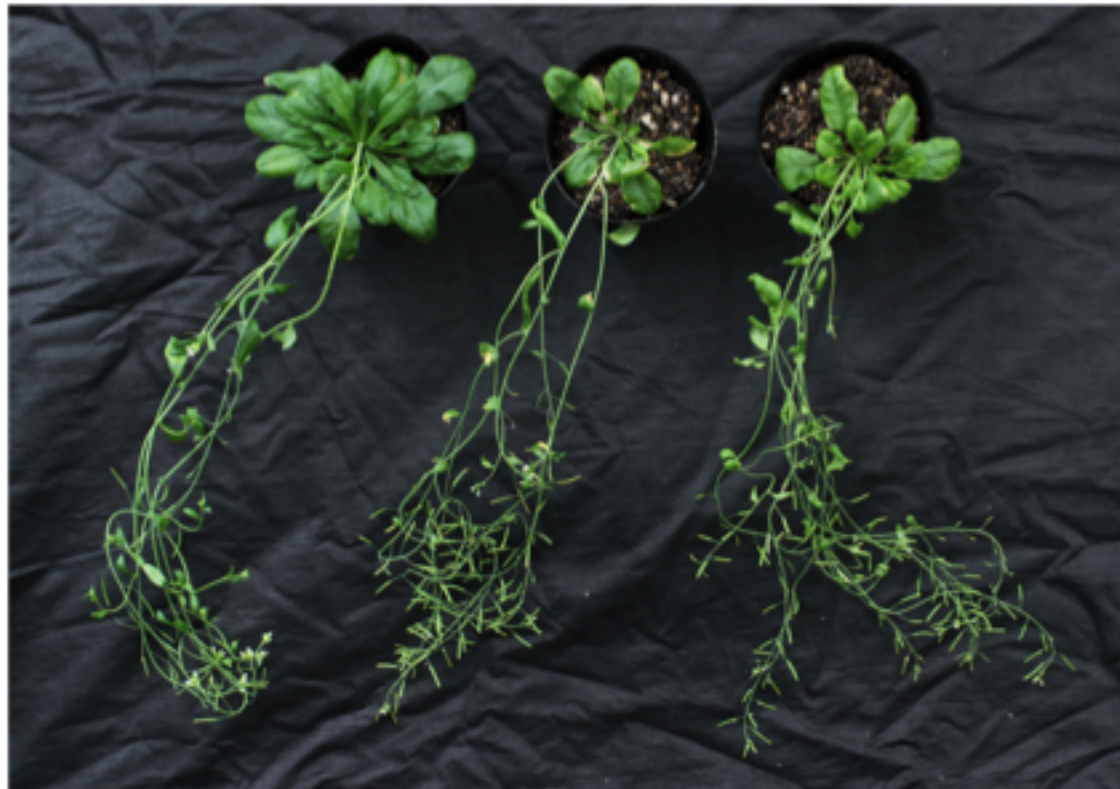

Supplement: Supplementary file 8 — Figure S7. Growth of representative ups1 and ups2 mutants. Plants were grown at 23 °C in soil for 7 weeks under long-day conditions. (PDF 257 kb) [file 12870_2018_1491_MOESM8_ESM.pdf]
