# Supplementary material for: Disruption of ureide degradation affects plant growth and development during and after transition from vegetative to reproductive stages
Source: BMC Plant Biol. 2018 Nov 20;18:287. doi: 10.1186/s12870-018-1491-2 (PMC6245725; doi:10.1186/s12870-018-1491-2)
Supplement: Supplementary file 3 — Table S1. Leaf morphometrics of aln and aah mutants. (DOCX 26 kb) [file 12870_2018_1491_MOESM3_ESM.docx]

**Table S1** Leaf morphometrics of *aln* and *aah* mutants

| Character (mm) | Wild-type | *aln-1* | *aln-2* | *aah* |
| --- | --- | --- | --- | --- |
| Leaf length | 31.2 ± 0.9 | 26.6 ± 1.2** | 27.8 ± 1.0* | 27.0 ± 1.2** |
| Leaf width | 15.8 ± 0.4 | 14.8 ± 0.5 | 14.9 ± 0.4 | 14.7 ± 0.7 |
| Petiole length | 18.0 ± 0.6 | 12.4 ± 0.6*** | 14.8 ± 0.5*** | 9.9 ± 0.8*** |

The biggest leaves in individual 5-week-old plants were selected visually.

Values are means ± standard error (*n* ≥ 21).

Significant differences using two-tailed *t*-tests based on the linear model at: *, *P* < 0.05; **, *P* < 0.01; ***, *P* < 0.001.
