# Supplementary material for: Disruption of ureide degradation affects plant growth and development during and after transition from vegetative to reproductive stages
Source: BMC Plant Biol. 2018 Nov 20;18:287. doi: 10.1186/s12870-018-1491-2 (PMC6245725; doi:10.1186/s12870-018-1491-2)
Supplement: Supplementary file 9 — Table S2. Composition of liquid culture media with low (2 mM) and standard (20 mM) concentrations of inorganic nitrogen. (DOCX 28 kb) [file 12870_2018_1491_MOESM9_ESM.docx]

**Table S2** Composition of liquid culture media with low (2 mM) and standard (20 mM) concentrations of inorganic nitrogen (N).

| Chemical | 2 mM N  (mg l^–1^) | 20 mM N  (mg l^–1^) |
| --- | --- | --- |
| NH_4_NO_3_ | 80 | 800 |
| KCl | 700 | 700 |
| CaCl_2_·2H_2_O | 220 | 220 |
| MgSO_4_·7H_2_O | 185 | 185 |
| KH_2_PO_4_ | 85 | 85 |
| H_3_BO_3_ | 3.09 | 3.09 |
| MnSO_4_·7H_2_O | 12.05 | 12.05 |
| ZnSO_4_·5H_2_O | 4.3135 | 4.3135 |
| KI | 0.415 | 0.415 |
| Na_2_MoO_4_·2H_2_O | 0.121 | 0.121 |
| CuSO_4_·5H_2_O | 0.0125 | 0.0125 |
| CoCl·6H_2_O | 0.01185 | 0.01185 |
| Na_2_-EDTA | 18.65 | 18.65 |
| FeSO_4_·7H_2_O | 13.9 | 13.9 |
| Thiamine·HCl | 0.25 | 0.25 |
| Nicotinic acid | 0.25 | 0.25 |
| Pyridoxin·HCl | 0.25 | 0.25 |
| Glycine | 1 | 1 |
| *myo*-Innositol | 50 | 50 |

The pH was adjusted to 5.6 with 1 M KOH.
