# Supplementary material for: Disruption of ureide degradation affects plant growth and development during and after transition from vegetative to reproductive stages
Source: BMC Plant Biol. 2018 Nov 20;18:287. doi: 10.1186/s12870-018-1491-2 (PMC6245725; doi:10.1186/s12870-018-1491-2)
Supplement: Supplementary file 10 — Table S3. Primers used in this study. (DOCX 32 kb) [file 12870_2018_1491_MOESM10_ESM.docx]

**Table S3. Primers used in this study**

| AGI^a^ | Gene  symbol^b^ | Direction | Primer sequence | Use^c^ |
| --- | --- | --- | --- | --- |
| At4g20070 | *AAH* | Forward | 5’-CTTTTGTGCTCCATTGAACGAAAGC-3’ | RT-qPCR |
|  |  | Reverse | 5’-AACAACATTCCCACCTTGGTTAAGTGT-3’ | RT-qPCR |
| At3g18780 | *ACT2* | Forward | 5’-ACCGTATGAGCAAAGAAATCAC-3’ | RT-qPCR |
|  |  | Reverse | 5’-GAGGGAAGCAAGAATGGAAC-3’ | RT-qPCR |
| At4g04955 | *ALN* | Forward | 5’-CCTGGTCTCATTGATGTGCATGTTC-3’ | RT-qPCR |
|  |  | Reverse | 5’-TGTTTTTCGCAGCTTCAATCTTGAGT-3’ | RT-qPCR |
| At2g28390 | *MON1* | Forward | 5’-AACTCTATGCAGCATTTGATCCACT-3’ | RT-qPCR |
|  |  | Reverse | 5’-TGATTGCATATCTTTATCGCCATC-3’ | RT-qPCR |
| At2g29350 | *SAG13* | Forward | 5’-CACAACTCCTTTAAGTAACG-3’ | RT-qPCR |
|  |  | Reverse | 5’-TTATGGCATAGTCTTGAAGG-3’ | RT-qPCR |
| At4g27960 | *UBC9* | Forward | 5’-TCACAATTTCCAAGGTGCTGC-3’ | RT-qPCR |
|  |  | Reverse | 5’-TCATCTGGGTTTGGATCCGT-3’ | RT-qPCR |
| At2g03590 | *UPS1* | Forward | 5’-TCATTGCTTCTCTAGAAACCCA-3’ (F1) | PCR genotyping |
|  |  | Reverse | 5’-TCTGCTCGGACCTATTTCTCC-3’ (R1) | PCR genotyping |
|  |  | Forward | 5’-GGATCCATTGAAGGAAGAGTTAC-3’ | qRT-PCR and |
|  |  |  |  | semi-qRT-PCR |
|  |  | Reverse | 5’-CCAATTGTTGTTAGCAAAGCC-3’ | qRT-PCR and |
|  |  |  |  | semi-qRT-PCR |
| At2g03530 | *UPS2* | Forward | 5’-GGTATCGTGCTTAGCCTCGGGAATC-3’ (F2) | PCR genotyping |
|  |  | Reverse | 5’-GCTGGAGAGAAGAGGGAGAAAC-3’ (R2) | PCR genotyping |
|  |  | Forward | 5’-GACTGGAATGGTCGTTACTGG-3’ | qRT-PCR and |
|  |  |  |  | semi-qRT-PCR |
|  |  | Reverse | 5’-AGCCTGAACGGAGTCAGCA-3’ | qRT-PCR and |
|  |  |  |  | semi-qRT-PCR |
| At4g34890 | *XDH1* | Forward | 5’-TGATGTTGGACAAATAGAAGGAGCGTTT-3’ | qRT-PCR |
|  |  | Reverse | 5’-TATTCGGATTCCCCTTGAGAAGCGAAACA-3’ | qRT-PCR |
| At1g78900 | *VHA-A* | Forward | 5’-ATGCCGGCGTTTTACGGAGG-3’ | Semi-qRT-PCR |
|  |  | Reverse | 5’-ATTTCCCAATATTCCTGGCC-3’ | Semi-qRT-PCR |
| − | T-DNA | − | 5’-TGGTTCACGTAGTGGGCCATCG-3’ (LBa1) | PCR genotyping |
| − | T-DNA | − | 5’-GCCTTTTCAGAAATGGATAAATAGCCTTGCTTCC-3’ | PCR genotyping |
|  |  |  | (SAIL LB1) |  |

^a^ *Arabidopsis thaliana* gene identifier code assigned by the Arabidopsis Genome Initiatives (AGI; https://www.arabidopsis.org/portals/nomenclature/).

^b^ Gene symbol as provided by TAIR (http://www.arabidopsis.org/) except T-DNA, which refers to the left border of T-DNA of *Agrobacterium tumefaciens*.

^C^ qRT-PCR, reverse transcription-quantitative polymerase chain reaction; semi-qRT-PCR, semi-quantitative reverse transcription-polymerase chain reaction.
